# Stimuli‐Responsive Supramolecular Biomaterials for Cancer Theranostics

**DOI:** 10.1002/advs.202515860

**Published:** 2026-01-07

**Authors:** Wenting Hu, Binglin Ye, Guocan Yu, Feihe Huang, Zhengwei Mao

**Affiliations:** ^1^ Department of Hepatobiliary and Pancreatic Surgery, The Second Affiliated Hospital Zhejiang University School of Medicine Hangzhou Zhejiang 310009 China; ^2^ MOE Key Laboratory of Macromolecular Synthesis and Functionalization, Department of Polymer Science and Engineering Zhejiang University Hangzhou Zhejiang 310027 China; ^3^ Key Laboratory of Precision Diagnosis and Treatment for Hepatobiliary and Pancreatic Tumor of Zhejiang Province The Second Affiliated Hospital of Zhejiang University Hangzhou Zhejiang 310009 China; ^4^ Research Center of Diagnosis and Treatment Technology for Hepatocellular Carcinoma of Zhejiang Province The Second Affiliated Hospital of Zhejiang University Hangzhou Zhejiang 310009 China; ^5^ Clinical Medicine Innovation Center of Precision Diagnosis and Treatment for Hepatobiliary and Pancreatic Disease Zhejiang University Hangzhou Zhejiang 310009 China; ^6^ Clinical Research Center of Hepatobiliary and Pancreatic Diseases of Zhejiang Province, The Second Affiliated Hospital of Zhejiang University Hangzhou Zhejiang 310009 China; ^7^ Cancer Center Zhejiang University Hangzhou Zhejiang 310009 China; ^8^ Key Laboratory of Bioorganic Phosphorus Chemistry & Chemical Biology Department of Chemistry Tsinghua University Beijing 100084 China; ^9^ Stoddart Institute of Molecular Science Department of Chemistry Zhejiang University Hangzhou Zhejiang 310027 China; ^10^ Zhejiang‐Israel Joint Laboratory of Self‐Assembling Functional Materials ZJU‐Hangzhou Global Scientific and Technological Innovation Center Zhejiang University Hangzhou Zhejiang 311215 China

**Keywords:** cancer theranostics, smart materials, stimuli‐responsive, supramolecular chemistry, tumor microenvironment

## Abstract

The ultimate goal of cancer theranostics is to deliver imaging agents and therapeutic cargo to tumor sites when and where they are required. “Smart” systems─including targeted and tailored releases with excellent spatial, temporal, and dosage control─should be developed. Supramolecular interactions with dynamic, reversible, and directional features enable the design of biomaterials with ordered architectures, tailored morphologies, multiple types of cargo, and controllable functions. Such supramolecular biomaterials are good candidates for oncology applications, as they optimize therapeutic efficacy while minimizing systemic adverse effects. This review summarizes recent advances in intelligent supramolecular biomaterials, with a specific focus on their spatiotemporal control in cancer theranostics. These biomaterials exploit endogenous and exogenous stimuli to trigger their morphological transformation. In particular, their construction approach and working mechanism, which are crucial in guiding tumor theranostics, are elaborated. Furthermore, the significant challenges in clinical translation and future perspectives for such biomaterials are discussed.

## Graphical abstract

The ultimate goal of cancer theranostics is to get imaging agents and therapeutic cargo to tumor sites when and where they are required. “Smart” systems should be developed. This review discusses the characteristics of physiological stimuli, types and action modes of external stimuli, construction approaches and working principles, as well as challenges and perspectives of stimuli‐responsive supramolecular biomaterials, with a particular emphasis on their role in achieving precise spatiotemporal control in cancer theranostics.

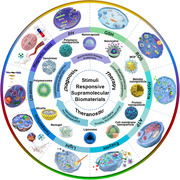

## 1 Introduction

Cancer arises as a result of a single cell that begins to behave strangely, to proliferate uncontrollably, and, ultimately, to invade or spread to adjacent tissues. As a significant public health issue with high morbidity and mortality worldwide, the development of advanced methods for the prevention, diagnosis, and treatment of tumors is of paramount importance [1]. Currently, the vast majority of patients with cancer are treated via surgery, chemotherapy, or radiotherapy to eradicate tumors. However, it is a rather difficult aim due to the complicated multifactorial etiology and pathological microenvironment of cancer, as well as individual variations.

Cancer theranostics ingeniously combines imaging reagents and therapeutic cargo in a single formulation, allowing for the evaluation of their biodistribution, the determination of their mechanism of action, and the monitoring of treatment progression in real‐time. The ultimate goal is to deliver imaging agents and therapeutic cargo to the tumor sites when and where they are needed. Unfortunately, the overall efficacy of the theranostics is undercut by on‐target activity in normal cells, eventually resulting in severe adverse effects. “Smart” theranostics systems, which facilitate specific targeting and tailored release with outstanding spatial, temporal, and dosage control, should be developed. To acquire such capabilities, intelligent stimuli‐responsive systems have been engineered to respond to endogenous and exogenous stimuli at the target site, enabling the precise delivery of drugs, genes, proteins, antibodies, or diagnostic reporter molecules to combat or monitor the tumor.

Supramolecular self‐assembly, which is a ubiquitous phenomenon in living organisms, refers to the spontaneous formation of intricate molecular architectures characterized by thermodynamic stability and a well‐organized arrangement of fundamental molecules or building blocks based on non‐covalent interactions [2, 3]. These interactions include hydrogen bonding, host–guest interactions, hydrophobic interactions, π–π stacking, electrostatic interactions, coordination interactions, and van der Waals forces [4, 5, 6, 7, 8]. Owing to the reversible nature of these interactions, supramolecular biomaterials offer several advantages over biomaterials based on other strategies: (i) Although separately non‐covalent interactions are weak, the sum and directionality of these interactions ensure the stability of the material in complex physiological environments. (ii) Various dimensions, morphology, physicochemical properties can be achieved through tailoring molecular compositions and motifs, including polymer‐based supramolecular materials (such as polymer nanoparticles, polymersomes, nanogels), biomimetic‐based supramolecular materials (such as cell‐membrane nanoparticles), supramolecular organic frameworks, etc. (iii) Most importantly, supramolecular biomaterials are inherently dynamic and tunable, enabling them to respond rapidly to specific stimuli and to regulate their architecture by inducing physical changes (such as swelling, shrinkage, charge conversion, phase transition, competitive binding, etc.), chemical changes (such as crosslinking, disassembly, degradation, dissociation, etc.), or a combination of both. (iv) Supramolecular materials lie in the potential to integrate various functionalities. Such versatility is important in cancer theranostics because multifunctionality typically stems from the incorporation of molecules with vastly different or even orthogonal properties, including bioactive components, active‐targeting ligands, and stimuli‐responsive moieties. Collectively, this supramolecular toolkit has proven useful and efficient in engineering stimuli‐responsive biomaterials for cancer theranostics.

Progress in supramolecular chemistry, materials engineering, and nanotechnology has supported the development of stimuli‐responsive supramolecular biomaterials. Such biomaterials not only allow for effective delivery to minimize off‐target toxicity but also offer spatiotemporal resolution and complex release properties, thereby maximizing the efficiency and safety of cancer theranostics. Physiological parameters from tumor cells, such as acidic environments, hypoxia, imbalanced redox homeostasis, and elevated adenosine triphosphate (ATP) and enzyme levels, as well as extracorporeal conditions like light, temperature, ultrasound, and magnetic fields, have been employed to perturb self‐assembled supramolecular biomaterials. The solubility, pharmacokinetics, and biological distribution of the cargo are altered by precisely controlling the duration and rate of their transport and release. By selecting and modifying these biomaterials, integrating their supramolecular forces, considering the physiological features of the site of action, and evaluating the pharmacodynamic and pharmacokinetic changes of payloads in response to various internal or external stimuli, cancer theranostics involving one or more regimens with distinct triggering mechanisms can be successfully applied.

**FIGURE 1:**
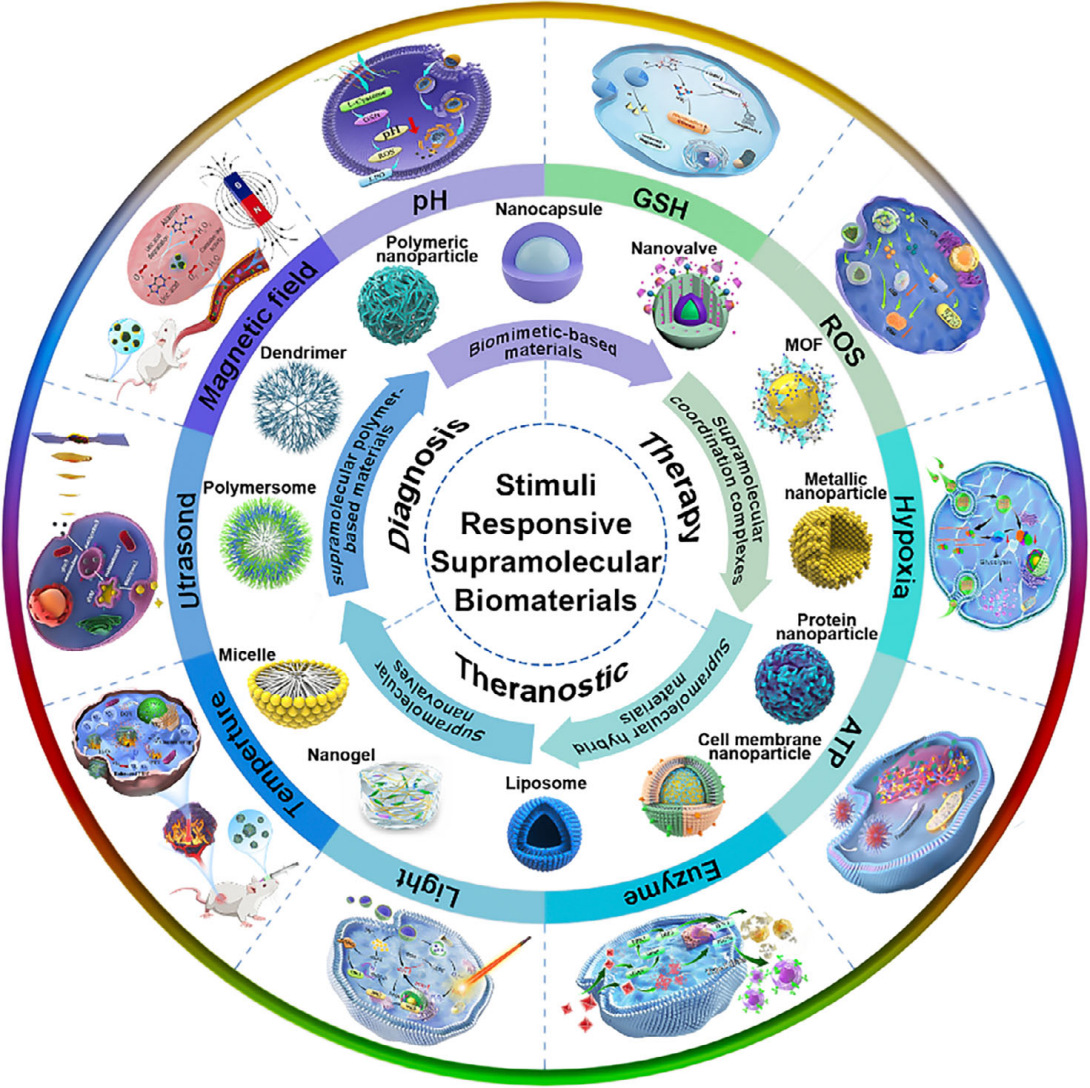
Stimuli‐responsive supramolecular biomaterials for cancer theranostics.

This review discusses the most significant advances of cancer theranostics in terms of spatiotemporal dynamics via supramolecular biomaterials with various physiological‐ or extracorporeal‐dependent properties (Figure 1). First, we summarize the characteristics of the response conditions in the tumor microenvironment and the types and modes of action of the external stimuli. Next, we emphasize various strategies that support and boost the therapeutic efficacy of supramolecular biomaterial‐based cancer theranostics. Notably, two remarkable aspects, the method by which stimuli‐responsive supramolecular assemblies are established and the working mechanism by which their structures respond to one or more particular stimuli, are elaborated. Then, in light of the clinical demand for cancer theranostics, we list the challenges in bringing innovative supramolecular biomaterials from the laboratory to the bedside. Finally, we provide a perspective toward future directions and opportunities for supramolecular biomaterials in cancer theranostics.

## 2 Various Stimuli

Abnormal physiological parameters are often associated with infections as well as diseases, including cancer and autoimmune diseases. Tumor characteristics, including acidity, hypoxic conditions, disrupted redox equilibrium, higher ATP levels, and enzyme overexpression, have emerged as attractive targets for designing responsive supramolecular biomaterials. External stimuli such as light, temperature, ultrasound, and magnetic field can also be applied to supramolecular biomaterials for improved therapeutic efficacy.

### 2.1 Physiological Stimuli to Mediate Cancer Theranostics

Tumors develop in complex and continuously evolving microenvironments in which the cancer cells interact dynamically and bidirectionally with their surrounding environment. The tumor microenvironment (TME) supports tumor cell survival and proliferation and facilitates metastasis and drug resistance [9, 10], which contribute to the development of cancer hallmarks. Relative to the entire TME, the specialized microenvironment appears to be a better target for tumor therapy, including a more acidic microenvironment, hypoxic status, redox disequilibrium (of glutathione (GSH) and reactive oxygen species (ROS)), high levels of ATP, and enzyme overexpression [11, 12]. These unique properties of the TME can be harnessed to design stimuli‐responsive supramolecular biomaterials, providing a promising strategy for cancer diagnostics and therapeutics. The specificity of these supramolecular platforms not only enhances therapeutic efficacy but also minimizes systemic side effects.

#### 2.1.1 Low pH

Acidity is a well‐known hallmark of solid tumors [11, 13, 14]. Tumor cells rely on the Warburg effect, in which energy is supplied via glycolysis rather than mitochondrial oxidative phosphorylation, inducing the generation and accumulation of lactic acid within the TME; the disorganized tumor vasculature prevents the effective diffusion of acid products, accelerating the accumulation of acid in the TME; carbonic anhydrase, an overexpressed enzyme in tumors, catalyzes the reversible hydration of carbon dioxide to produce carbonic acid, which is another non‐negligible source of acidification in the TME [15]. Consequently, the extracellular microenvironment is acidic (pHe 6.4–7.1) while the intracellular microenvironment is alkaline (pHi 7.1–7.8) within tumours [16, 17]. This pH gradient creates a perfect storm for cancer progression. In contrast, under physiological conditions, the pH of normal tissues typically ranges from 7.2 to 7.4 (pHe ∼7.4 and pHi ∼7.2), which is maintained through active acid/base transport processes from the cell membranes into the extracellular spaces [18, 19]. In addition, the endosomal–lysosomal system exhibits a pH value of 5.0–6.2 (early endosome, pH 5.9–6.2; late endosome/lysosome, pH 5.0–5.5) in both normal and tumor cells, which is a characteristic that is not unique to the TME. Tumor acidosis can be neutralized by bicarbonate transporters or proton pump inhibitors [20], inhibited by lactate dehydrogenase inhibitors that block the conversion of pyruvate to lactic acid, or harnessed to engineer intelligent supramolecular materials [21] that exhibit marked physiochemical responses to such delicate changes in pH value.

#### 2.1.2 Hypoxia

Low oxygen tension, a common feature of solid tumors, plays a pivotal role in tumor progression, angiogenesis, and metastasis [22]. Tumor hypoxia arises from the mismatch between oxygen consumption for the proliferation of cancer cells and the inadequate supply by the dysfunctional microvasculature. The partial pressure of oxygen in tumor cells falls below 10 mmHg, compared to 40–60 mmHg in most healthy tissue (an O_2_ partial pressure of 15 mmHg corresponds to approximately 2% O_2_ in the gas–phase or 20 µm O_2_ in solution) [23, 24]. The extent of hypoxia varies with the tumor type, stage, size, location (surface or center) within the tumor tissue, and the distance to tumor microvessels. Some areas of a tumor may be anoxic (0 mmHg) or severely hypoxic (as low as 2.5 mmHg) [25]. The hypoxic TME can severely impair the effect of oxygen‐related therapies such as chemodynamic therapy, photodynamic therapy (PDT), sonodynamic therapy, radiotherapy, and immunotherapy. For instance, in PDT, the excited photosensitizer converts triplet oxygen to cytotoxic singlet oxygen to damage tumor cells [26]; in radiotherapy, oxygen, a potent radiosensitizer, reacts with ionizing radiation‐induced free radicals to cause irreversible DNA damage and enhances cell‐killing efficacy by disrupting DNA self‐repair [27, 28]; in immunotherapy, tumor hypoxia poses a substantial obstacle by causing overexpression of hypoxia‐inducible factors, primarily hypoxia‐inducible factor‐1α, which regulate tumor angiogenesis and modulate the expression of multiple angiogenic growth factors [29, 30, 31]. Cancer theranostics can be improved by developing hypoxia‐responsive supramolecular biomaterials [32], which can load therapeutic agents (e.g., hypoxia‐activated prodrug, inhibitor) [33, 34], oxygen transporters (e.g., perfluorocarbons) [35], gas‐generating substances (e.g., superoxide dismutase) [36], imaging agents, and targeting ligands [37].

#### 2.1.3 High‐Level GSH

GSH, a tripeptide with a γ‐amide bond and a thiol group, is the most widespread non‐protein thiol at millimolar concentrations in mammalian tissues. As an intracellular antioxidant, it plays an indispensable role in maintaining redox homeostasis through its reversible oxidation and reduction states. GSH concentrations in tumor cells can generally reach 0.5–10 mm, several times those of normal cells. Notably, cytoplasmic GSH concentrations are approximately 1000‐fold higher in tumor cells than in the blood or extracellular matrix (2–20 µm). This surge results from the conversion of oxidized glutathione into GSH via the catalysis of GSH reductase and nicotinamide adenine dinucleotide phosphate (NADPH) in the cytosol, which protects the tumor cell from oxidative stress‐induced damage. Therefore, GSH can serve as a sensitive and efficient target for the development of stimuli‐responsive delivery systems that utilize functional groups or chemical bonds with oxidation properties [38], GSH‐mediated depletion therapy [39], and GSH‐triggered detection via imaging probes activation [40].

#### 2.1.4 High‐Level ROS

ROS, which are extremely unstable and short‐lived, include radical species (such as superoxide anions (O_2_•^−^), peroxyl radicals (ROO•), nitric oxide (NO•), and nitrogen dioxide (NO_2_•)) and non‐radical species (such as peroxynitrite (ONOO^−^) and singlet oxygen (^1^O_2_)) [41, 42]. ROS, derived primarily from the mitochondrial respiratory chain and NADPH oxidase, profoundly influence the occurrence and progression of cancer. Genetic alterations and transformation of the energy–metabolism mode further drive ROS generation, leading to tumor invasion and metastasis. The H_2_O_2_ concentration in the TME can reach up to 100 µm, approximately three orders of magnitude higher than in normal cells (10–700 nm). Furthermore, ROS can induce immunogenic cell death via ROS‐mediated pyroptosis in various cancers, which offers a promising approach for boosting cancer immunotherapy [43]. Hence, the typically high ROS levels in the TME can be exploited by designing oxidation‐responsive supramolecular biomaterials to regulate tumor cell death (e.g., apoptosis [44], ferroptosis [45, 46], and pyroptosis [47, 48]) by enhancing cytotoxic ROS levels, or to treat inflammatory diseases by scavenging cytotoxic ROS levels [49, 50].

#### 2.1.5 High‐Level ATP

ATP is a universal and essential metabolite that participates in energy transduction and biological signaling [51]. Since ATP cannot be synthesized in the extracellular milieu, it is present in remarkable concentrations (5–10 mm) within cells and is rarely absent from the extracellular compartment of healthy tissue (estimated at 10–100 nm) [52, 53]. However, the extracellular ATP concentration in tumor cells is typically in the micromolar range (50–200 µm) owing to the release of intracellular ATP via cell death‐induced conditions [54, 55]. Based on these findings, ATP has emerged as a viable marker for the development of ATP‐driven supramolecular biomaterials, including ATP‐responsive, ATP‐triggered, or ATP‐fueled systems, for cancer treatment and theranostics.

#### 2.1.6 Overexpressed Enzymes

Enzymes are typically proteins, but they can also be RNA molecules. As biological catalysts, they participate in and drive, in a sustainable, mild, efficient, and specific manner, almost all chemical reactions involved in biological and metabolic processes. Enzymes such as hyaluronidases, caspases, γ‐glutamyl transpeptidases, matrix metalloproteinases, cathepsin B, heat shock proteins, lactate dehydrogenase, indoleamine 2,3‐dioxygenase 1, quinone oxidoreductase‐1, carbonic anhydrases, and esterases are overexpressed in the TME [56]. For instance, matrix metalloproteinase‐2, an important subtype of matrix metalloproteinases, is overexpressed by approximately 43‐fold in colorectal cancer cells [57]. Hyaluronidase, which degrades hyaluronic acid in the extracellular matrix to modulate the proliferation, invasion, and metastasis of cancer cells [58], exhibits greater overexpression in metastases than in primary tumors. Indoleamine 2,3‐dioxygenase 1, the rate‐limiting enzyme in the metabolism of tryptophan to kynurenine, causes tryptophan depletion and kynurenine accumulation [59], thereby contributing to the establishment of an immunosuppressive environment [60]. Quinone oxidoreductase‐1, a member of the NAD(P)H dehydrogenase (quinone) family, is overexpressed by 5–200 fold in various types of tumors [61]. These dysregulated enzymes are favorable targets for constructing enzyme‐triggered self‐assembly [62, 63], structural transition [64], and disassembly systems [65] for drug delivery and early cancer diagnostics.

### 2.2 Exogenous Stimuli to Modulate Cancer Theranostics

Endogenous physiological stimuli provide favorable targets for formulating strategies to achieve spatial control of therapeutic systems, although they lack the potential for temporal regulation. In particular, immediate or burst‐release mechanisms have disadvantages such as low absorption efficiency, short action time, and local toxicity.  Critically, smart supramolecular biomaterials offer spatiotemporal controllability, enabling their site‐specific localization with on‐demand release patterns over a defined period. To provide these capabilities, engineered supramolecular biomaterials respond to exogenous stimuli (such as light, temperature, ultrasound, and magnetic fields), enabling real‐time release of therapeutic agents and enhanced therapeutic efficacy in cancer treatment [66, 67, 68].

#### 2.2.1 Light

Light, as a clean energy source and noninvasive stimulus, has received significant attention in recent years. Photo‐induced reactions offer precise spatiotemporal control and do not involve the production of waste products compared to most chemical‐responsive systems. Owing to these advantages, many types of phototherapy have been designed, including those that utilize external sources (such as ultraviolet, visible, and near‐infrared light) or internal sources (such as chemiluminescence, electrochemiluminescence, and bioluminescence). Since ultraviolet and visible light have sufficient energy to cleave chemical bonds, they are appropriate for therapeutic delivery. Since near‐infrared light exhibits deep tissue penetration with negligible tissue damage, it is appropriate for PDT [69] and photothermal therapy (PTT) [70]. These modalities cause tumor regression via chemical and thermal damage, respectively. PDT‐mediated chemical damage arises from both type I ROS—generated in the electron or hydrogen transfer process with substrates [71] and type II ROS—generated in the singlet‐to‐triplet transition process of photosensitizers [72, 73]. PDT‐mediated thermal damage results from localized heating of cells [74].

#### 2.2.2 Temperature

Dysregulation of metabolic activities and alterations in blood perfusion can cause localized hyperthermia (reaching 40–42 °C) within the tumor and the body. Given these facts, temperature variations can be utilized to trigger sharp and nonlinear changes in the structure or function of at least one component within the material, thereby enabling the construction of stimuli‐responsive supramolecular delivery systems. Electromagnetic waves, ultrasound waves, and laser light have been used as heat sources in supramolecular nanoplatform‐based heat therapy [75]. According to the extent of the temperature increment, therapeutic heating can be classified as mild or hyperthermia. Mild heating (up to 41–43 °C) impairs key biological functions of cancer cells, including DNA synthesis, DNA repair, and autophagy [76], while hyperthermia (>50 °C) preferentially damages tumor cells by causing coagulation and protein denaturation [77]. Moreover, thermal therapy can promote immunogenic cell death, releasing damage‐associated molecular patterns and contributing to the establishment of an immunogenic TME that suppresses the primary tumor and its distant metastasis [78]. Thermal‐mediated supramolecular systems thus have great potential for use in clinical therapy.

#### 2.2.3 Ultrasound

Ultrasound refers to a mechanical wave with a period of 20 kHz to 20 MHz. The use of this frequency range is appealing because it is imperceptible to human ears, interacts safely with human tissue and organs, exhibits relatively low loss in the human body, and results in the rapid exchange of energy [79, 80]. A wide range of ultrasound‐responsive materials has been developed for tumor theranostics, which exhibit several pivotal benefits, including the capacity for deep tissue penetration (on the order of centimeters), ease of regulation (by adjusting the duty cycle, frequency, and duration), as well as precise localization of energy deposition [81, 82]. As ultrasound passes through tissues, it exerts thermal effects, acoustic–mechanical effects, and (via cavitation) chemical effects. Sonothermal potencies can be applied to generate local hyperthermia, leading to the destruction of the targeted cancer cells [83]; sonomechanical effects can cause pores to form in the cell membrane, enhancing the intracellular delivery of drugs or genes [84]; while sonodynamic therapy, which promotes ROS production through sonochemical activation, effectively induces damage to tumor cells [85].

#### 2.2.4 Magnetic Field

Magnetic fields, including high‐frequency fields (>100 kHz) and low‐frequency fields (<20 kHz), are among the most extensively studied exogenous stimuli in the field of cancer theranostics [86, 87]. The benefits of utilizing magnetic stimulation include its biocompatibility and deep tissue penetration. The efficacy of magnetic stimulation depends primarily on its frequency, duration, and intensity. These parameters can be tailored to the type and size of the tumor, as well as the individual characteristics of the patient, to provide personalized cancer treatment [88]. Meanwhile, magnetic‐assisted targeting, achieved by focusing an external magnetic field on the tumor site, markedly improves the accumulation of desired substances, particularly genes, drugs, proteins, and antibodies [89, 90]. Given these benefits, magnetically responsive supramolecular biomaterials have been widely investigated for use in cancer theranostics [91]. For instance, in magnetically controlled delivery, therapeutic agents can be guided and concentrated toward certain cancer targets and activated remotely for on‐demand release [92]. In magnetic‐mediated thermal therapy, tumor ablation is realized by localized heat dissipation of magnetic particles under a high‐frequency magnetic field [93]. In magnetic‐mediated dynamic therapy, magnetic biomaterials, which act as ROS generators via the Fenton reaction within cancer cells or in the TME, hold great promise for cancer immunotherapy via ROS‐triggered regulation of the immunological TME [94]. In magnetic resonance imaging, magnetic materials are used as contrast agents [95].

## 3 Stimuli‐Responsive Supramolecular Biomaterials

**FIGURE 2:**
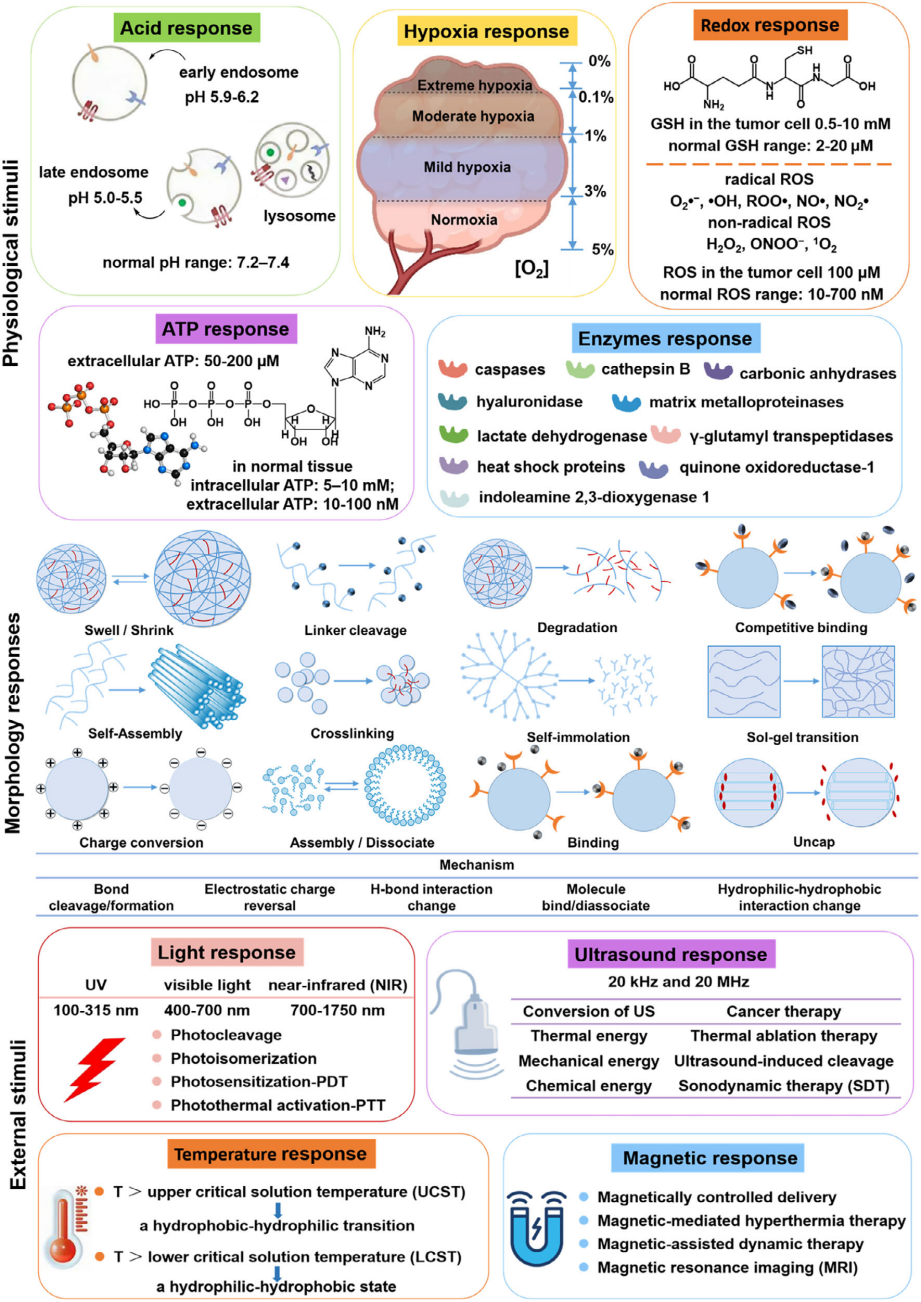
Response of supramolecular biomaterials to endogenous and exogenous stimuli for cancer theranostics. The figure illustrates diverse morphological changes in biomaterials triggered by acidic environments, hypoxia, redox imbalance, elevated ATP levels, enzyme overexpression, light, temperature, ultrasound, and magnetic fields.

The therapeutic formula, building blocks or scaffolds, and “load‐and‐release” smart units are integrated to construct environmental stimuli‐responsive supramolecular biomaterials that can respond differently toward internal stimuli (pH, hypoxia, GSH, ROS, ATP, and enzymes) and external triggers (light, temperature, ultrasound, and magnetic fields), achieving improved therapeutic efficacy [96]. Most notably, these materials can be described as “transformable”, in that their morphology can change in response to stimuli. Selecting the proper stimulus is central to achieving the desired tumor treatment and/or diagnosis. The ideal response behavior should be highly selective, sensitive, and controllable. A great variety of supramolecular biomaterials can be used for cancer theranostics. These include (1) supramolecular polymer‐based materials (e.g., polymeric nanoparticles, dendrimers, micelles, hydrogels) [97, 98]; (2) biomimetic‐based materials (e.g., cell membrane nanoparticles, liposomes) [99, 100]; (3) supramolecular coordination complexes based on organic ligands and metallic receptors (e.g., iron, platinum, palladium, rhodium, ruthenium, and iridium) [101, 102]; (4) supramolecular hybrid materials fabricated via self‐assembly of metal–organic frameworks, mesoporous silica nanoparticles, gold nanoparticles and iron oxide nanoparticles [103]; (5) supramolecular nanovalves comprising porous skeletons (hybrid) with lockable pore entrances [104]. Responses to stimuli, which can enhance cellular uptake, improve endosomal escape, or facilitate cargo release, can be based on physical changes (such as swelling, shrinkage, charge conversion, or competitive binding), chemical changes (including crosslinking, self‐assembly, degradation, dissociation, and phase transition), or a combination of both (Figure 2). The sensitivity to internal and external stimuli in non‐hybrid supramolecular biomaterials can be attributed to the cleavage or formation of bonds involving responsive functional groups [105, 106]. In hybrid supramolecular biomaterials, this sensitivity is further associated with magnetic, mechanical, and thermal responses [107, 108].

### 3.1 pH‐Responsive Supramolecular Biomaterials

**FIGURE 3:**
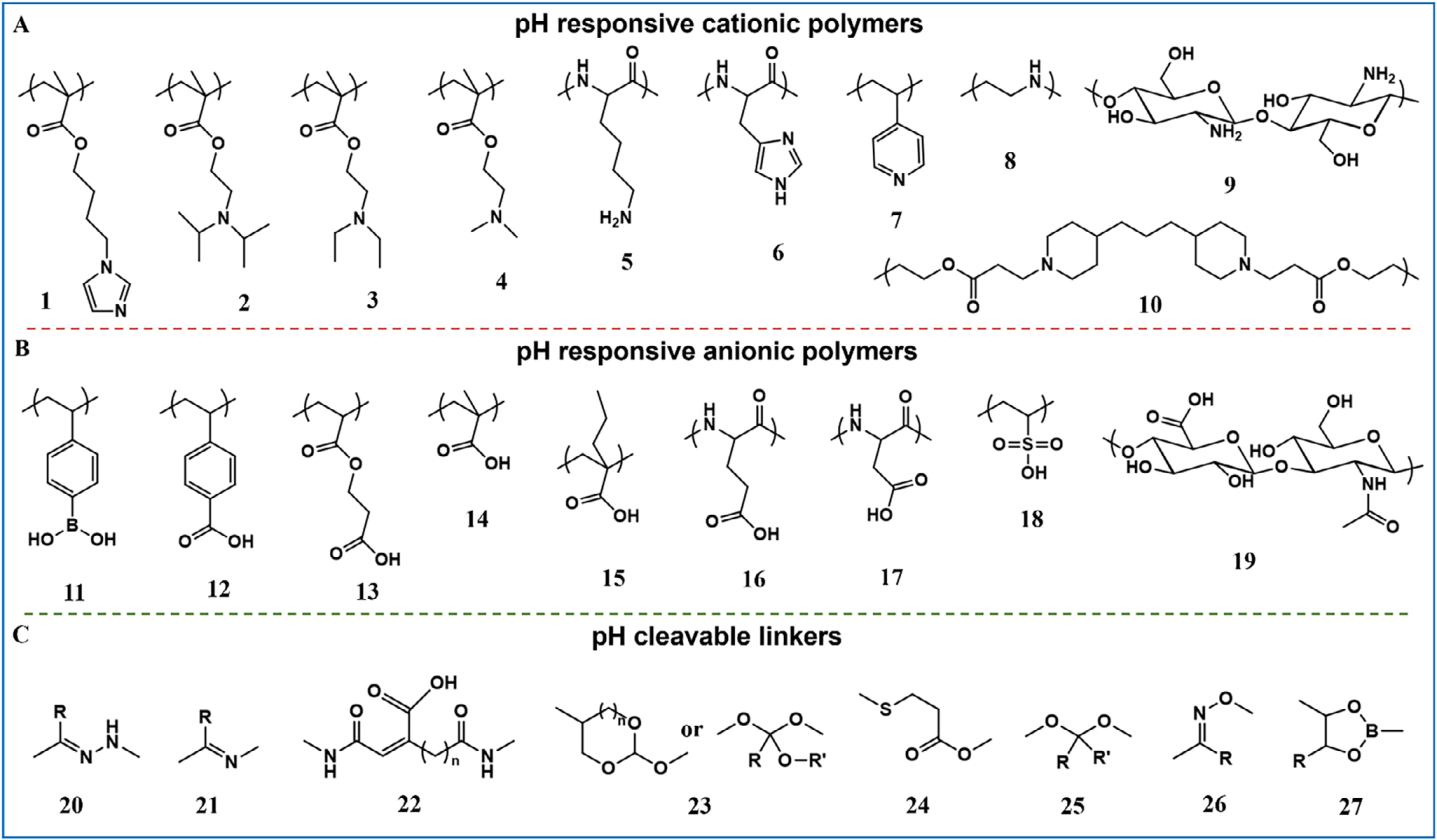
Summary of functional groups and linkers used in the field of pH‐responsive supramolecular biomaterials. (A) pH‐responsive cationic polymers include: (1) poly(4‐(1H‐imidazol‐1‐yl)butyl methacrylate (PImBuMA), (2) poly(2‐diisopropylaminoethyl methacrylate) (PDPAEMA), (3) poly(2‐diethylaminoethyl methacrylate) (PDEAEMA), (4) poly(2‐dimethylaminoethyl methacrylate) (PDMAEMA), (5) poly(lysine) (PLys), (6) poly(histidine) (PHis), (7) poly(4‐vinylpyridine) (PVP), (8) poly(ethylenimine) (PEI), (9) chitosan, and (10) poly(β‐amino ester) (PBAE). (B) pH‐ responsive anionic polymers include: (11) poly(vinylphenylboronic acid) (PVPBA), (12) poly(4‐vinylbenzoic acid) (PVBA), (13) poly(2‐carboxyethyl acrylate) (PCEA), (14) poly(acrylic acid) (PAA), (15) poly(2‐propylacrylic acid) (PPAA), (16) poly(glutamic acid) (PGA), (17) poly(aspartic acid) (PAsA), (18) poly(vinylsulfonic acid) (PVSA), and (19) hyaluronic acid. (C) pH‐cleavable linkers include: (20) hydrazine, (21) imine, (22) maleic acid amide, (23) ortho ester, (24) β‐thiol‐propionate, (25) acetal/ketal, (26) oxime, and (27) boronate ester.

The acidity of the TME has inspired the broad development of pH‐responsive supramolecular biomaterials for cancer treatment. Two primary methods are used to develop these biomaterials [109, 110]. The first method involves introducing acid‐cleavable chemical linkages into the therapeutic/diagnostic units or supramolecular scaffolds. Once the supramolecular biomaterial reaches the tumor cell, the acid‐labile linkages are cleaved, resulting in the dissociation, disassembly, and swelling of its structure to release the cargo or alter fluorescence. The supramolecular biomaterials involved in this method mainly include acid‐labile polymer–drug conjugates, crosslinked polymers, peptide conjugates, hybrid nanoparticles, metal–organic frameworks, and supramolecular nanovalves. The most frequently used pH‐sensitive chemical bonds involve the acetal/ketal group, the ortho ester moiety, the imine bond, the hydrazone bond, the ether (vinyl ether) bond, the β‐thiopropionate moiety, the 2,3‐dialkymaleic anhydrides structure [111], and the cis‐aconityl group. The second method involves selecting supramolecular biomaterials with functional groups capable of pH‐dependent protonation or ionization. The pH‐triggered charge conversion exerts a profound influence on the hydrophilic–hydrophobic equilibrium within the architecture of biomaterials [112, 113]. Such supramolecular biomaterials involved in this method mainly include amphiphilic polymers [114], polypeptides, micelles, liposomes, nanogels/microgels, and hybrid materials, facilitating the desired therapeutic effect (Figure 3). [115, 116, 117, 118]

**FIGURE 4:**
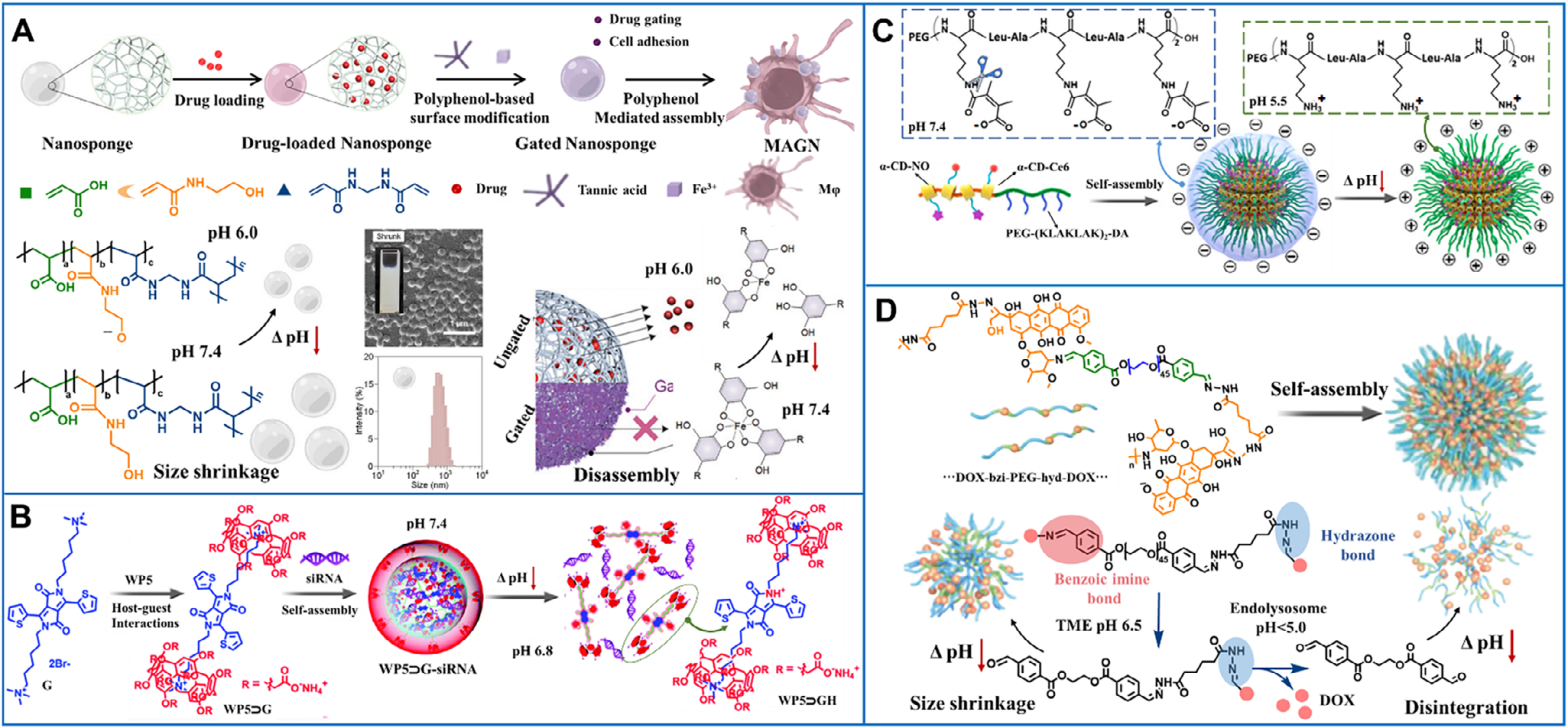
(A) Schematic illustration of the synthesis and pH‐induced disintegration of nanosponges capped with metal‐phenolic supramolecular gatekeepers (MAGN). Adapted with permission [120]. Copyright 2023, WILEY‐VCH Verlag GmbH & Co. KGaA, Weinheim. (B) Schematic illustration of the synthesis of WP5⊃G–siRNA supramolecular nanovesicles and pH‐induced siRNA release for intracellular therapy. Adapted with permission [121]. Copyright 2022, Royal Society of Chemistry. (C) Schematic illustration of the synthesis of supramolecular nanocarriers (α‐CD‐Ce6‐NO‐DA) and their pH‐responsive surface‐charge switching. Adapted with permission [123]. Copyright 2020, American Chemical Society. (D) Schematic illustration of pH‐induced size transformation of supramolecular nanoprodrug (PDNP)‐mediated cancer chemo‐immunotherapy. Adapted with permission [124]. Copyright 2022, WILEY‐VCH Verlag GmbH & Co. KGaA, Weinheim.

Supramolecular biomaterials containing acid‐protonated units can undergo structural changes (e.g., expansion, shrinkage, disassembly, assembly [119]) to release their therapeutic substances or aggregate on the cancer cell surfaces. pH‐responsive supramolecular nanosponges were synthesized, whose ionizable carboxyl acid groups could accept and donate protons upon pH change. After encapsulating a model chemotherapeutic drug, doxorubicin, and installing gatekeepers, the resulting gated nanosponges were assembled on bioactive tumor‐homing macrophages via polyphenol‐mediated interactions, referred to as MAGN. This system could be directed to tumors via cellular chemotaxis, where the pH‐responsive gatekeepers enabled efficient drug release, thus suppressing tumor progression and lung metastases in vivo (Figure 4A) [120]. pH‐sensitive supramolecular nanovesicles with a core–shell structure were prepared from pillararene‐based hosts and pH‐responsive guests for siRNA delivery. This hierarchical self‐assembly, driven by multiple interactions, guaranteed high loading, stability, and bioactivity of the siRNA. In the acidic TME, protonation triggered the complete collapse of the nanovesicles, releasing the siRNA and lighting up a fluorescent signal to trace the outcomes of gene therapy (Figure 4B) [121]. In addition, pH‐responsive supramolecular nanomedicines were constructed via hydrogen‐bonding and metal‐coordination interaction of thiourea motifs, enabling synergistic chemo/chemodynamic therapy. The low pH in the TME disrupted the double H‐bonds between thiourea motifs and carbonyl groups of doxorubicin, giving rise to its release for synergistic cancer treatment [122].

Alternatively, pH‐sensitive supramolecular biomaterials can be engineered by utilizing acid‐cleavable bonds that break under acidic conditions. A representative nanocarrier (α‐CD‐Ce6‐NO‐DA) was self‐assembled via host‐guest interactions. Acidic TME‐triggered amide bond cleavage reversed the nanocarriers’ surface charge, promoting biofilm penetration; the peroxynitrite, which arose from the reaction of PDT‐generated ROS with GSH‐mediated nitric oxide, provided a potent bactericidal effect. Thus, this supramolecular biomaterial, which integrated pH‐mediated surface‐charge shifting, nitric oxide‐mediated GSH‐depletion, and Chlorin e6‐mediated PDT, demonstrated superior anti‐infection activity against biofilms (Figure 4C) [123].

The pH gradient (pH ∼ 4–6) of the endosomal–lysosomal system can be exploited to achieve effective drug accumulation in organelles. A supramolecular prodrug with an acid‐cleavable backbone was developed for pyroptosis‐associated immunotherapy. Its nanoparticles exhibited a two‐step acid response, involving initial protonation of benzoic imine bonds at a mildly acidic pH and subsequent fracture of hydrazone bonds in the more acidic endolysosome. This process enabled precise nuclear delivery of doxorubicin, which effectively provoked gasdermin E‐mediated pyroptosis to boost antitumor immunity (Figure 4D) [124].

### 3.2 Hypoxic‐Responsive Supramolecular Biomaterials

**FIGURE 5:**
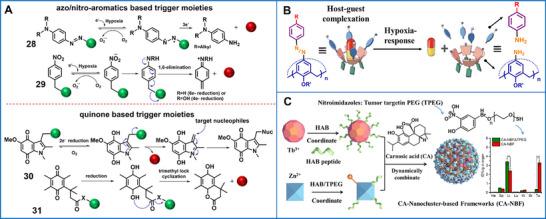
(A) Summary of functional groups used in the field of hypoxic‐responsive supramolecular biomaterials: (28) azoaromatics, (29) nitroaromatics, (30) indolequinone, (31) benzoquinone. Un‐activated/activated cargo = blue/red. (B) Schematic illustration of the supramolecular radiotherapy based on hypoxic‐responsive azocalixarene‐based host−guest delivery systems and the corresponding enhanced therapeutic efficiency mechanism. Adapted with permission [132]. Copyright 2022, WILEY‐VCH Verlag GmbH & Co. KGaA, Weinheim. (C) The composition of carnosic‐nanocluster‐based frameworks in response to hypoxia. Adapted with permission [133]. Copyright 2021, Elsevier.

Hypoxia, which is probably a more fundamental characteristic of many diseases, is a privileged target in personalized medicine, especially in the realm of cancer. Hypoxic‐responsive supramolecular nanoplatforms exploit various up‐regulated bioenzymes associated with reduction reactions or electron donation (including azoreductase, nitroreductase, methionine synthase reductase, and inducible nitric synthase), rather than low levels of oxygen as a trigger [33, 125, 126]. Such supramolecular biomaterials targeting hypoxic microenvironments encompass supramolecular polymers [127] and nanovesicles [128], which can be activated to release diverse therapeutic or bioimaging reagents [129] or can act as oxygen‐mimetic radiosensitizers. Furthermore, hypoxic‐responsive hybrid supramolecular biomaterials—constructed from metal–organic frameworks, inorganic nanoparticles, and supramolecular gatekeepers—are generally employed to synergize with chemotherapy and radiotherapy, PDT, or sonodynamic therapy. In these applications, tumor hypoxia must be explicitly considered, whether by alleviation, exploitation, or deliberate disregard, since it otherwise impairs therapeutic efficacy [130]. Typical hypoxic‐responsive groups are azobenzene, N‐oxides, nitro‐groups, quinones, and transition‐metal complexes (e.g., Co (III), Ru (III), and Pt (II, IV)) (Figure 5A) [126, 131]. In such supramolecular biomaterials, the introduction and response mechanisms for hypoxic‐responsive groups varies greatly depending on the selected hypoxic‐sensitive group.

The azobenzene group can be easily conjugated to artificial macrocyclic receptors, particularly calixarene [134]. Guo's group recently developed a series of hypoxic‐responsive azocalixarene‐based drug delivery systems utilizing host–guest interactions [135]. In these hypoxia‐responsive drug carriers, the anchoring of azobenzene groups on the upper rim of calixarenes expanded the area of the hydrophobic cavities, allowing high‐affinity interactions with therapeutic agents. On the basis of such a system, sugar groups and cationic/anionic groups (such as quaternary ammonium, sulfonate, and carboxyl groups) were selected to enhance solubility and bring additional functional sites; azocalix[n]arenes with different numbers of phenolic units (n = 4, 5, 6, 8) were introduced to match the various sizes of the drug molecules; galactose and lactose groups were incorporated into the molecular design to improve active targeting [135]. Under the hypoxic microenvironment, the azobenzene group can be specifically reduced into two separate aniline groups and isolated from calixarene (Figure 5B), structurally altering the supramolecular delivery system and triggering the release of therapeutic substances [132], which may comprise two drugs in different proportions [136], imaging agents [137], or albumin‐based nanosystems [138].

Most bioreductive supramolecular biomaterials feature quinones and nitro groups as inducers. Under hypoxic conditions, quinones are reduced either to semiquinone radicals via a one‐electron reduction pathway, which enhances the generation of ROS and improves oxidative stress; or to hydroquinones via a two‐electron reduction enzyme, which exhibits high cytotoxicity by mediating DNA cross‐linking [139]. In hypoxic tumor cells, nitro groups are reduced sequentially into nitroso, hydroxylamino, and amino groups via bioreduction reactions [140]. Based on this, quinones and nitroaromatic groups bond chemically with therapeutic reagents, supramolecular skeletons, or the surfaces of mesoporous materials, to be subsequently integrated into biomaterials via supramolecular interactions for cancer treatment and bioimaging [141]. Furthermore, drugs that directly select quinone as a drug‐core scaffold provide another approach for fabricating hypoxic‐responsive units [142]. A therapeutic framework based on carnosic acid nanoclusters was developed for tumor‐specific immunotherapy. Carnosic acid enabled peptide targeting and oligomerization, driving its 3D ordered self‐assembly with the Tb‐peptide complex (Tb‐HAB) and Zn‐peptide complex (Zn‐HAB‐TPEG) conjugated with nitroimidazole‐modified sulfhydryl polyethylene glycol (TPEG). This system achieved high loading, hypoxia‐targeting, GSH‐responsive disassembly, and satisfactory stability (Figure 5C) [133].

Transition metal‐based prodrugs constitute one of the most promising approaches for targeting tumor hypoxia [143]. Generally, coordination between metal complexes and cytotoxic agents may convert cytotoxic agents into less active prodrug forms with relatively low cytotoxicity and stabilize metal complexes with relatively high oxidation states. Upon bioreduction, this steady‐state may be impaired by reducing the metal complexes to a lower oxidation state, resulting in the release of active cytotoxic substances. Inspired by this, dual‐emissive Pt‐based supramolecular complexes were prepared for hypoxia imaging via the self‐assembly of an anthracene‐based blue fluorophore, a metal–porphine phosphorescent ligand, and a metal acceptor. Following encapsulation in an amphiphilic polymer, the resulting material enabled hypoxia detection through a selective increase in phosphorescence intensity under intracellular low‐oxygen conditions, while the blue fluorescence remained unchanged [144].

### 3.3 GSH‐Responsive Supramolecular Biomaterials

**FIGURE 6:**
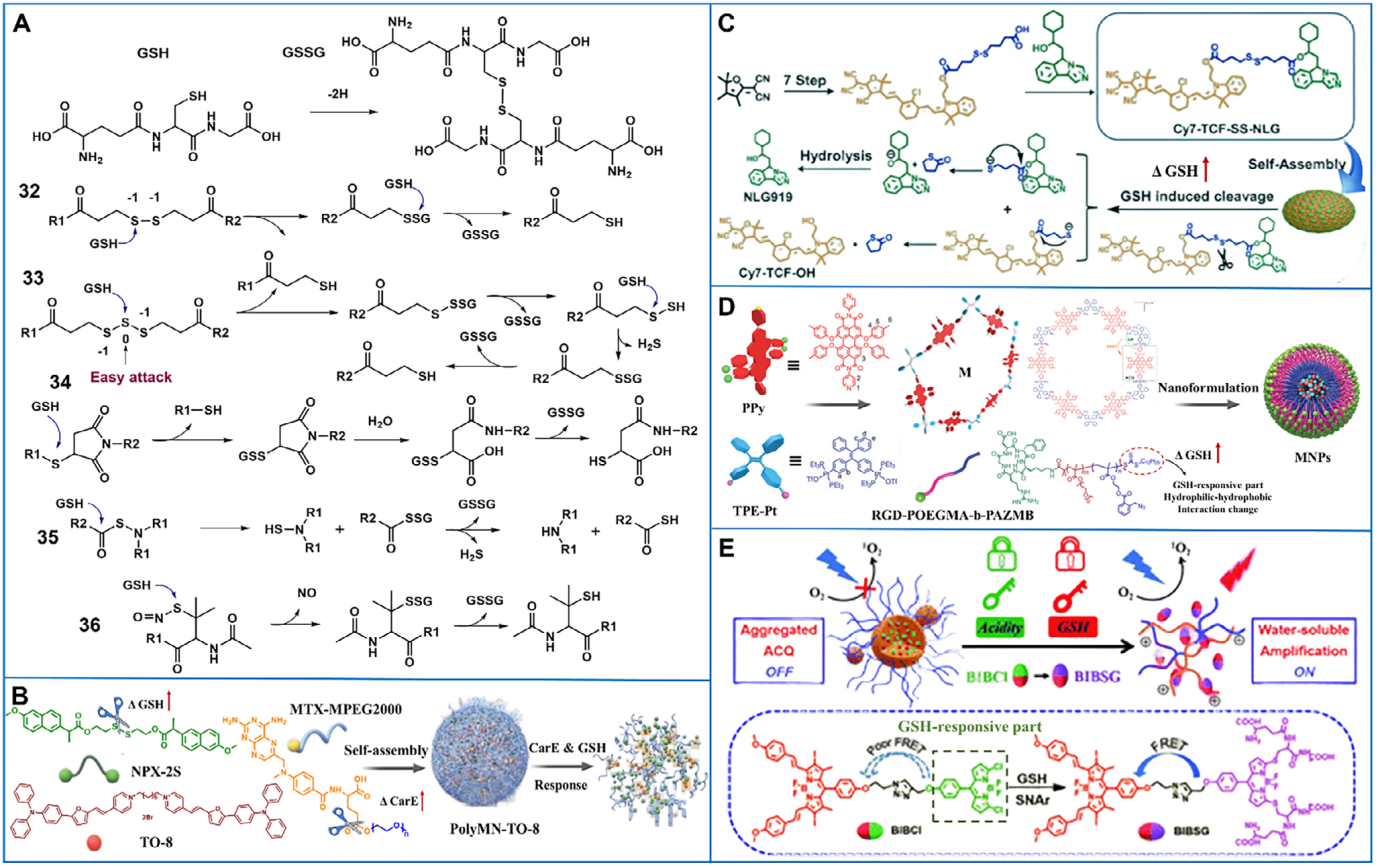
(A) Summary of the functional groups used in the field of GSH‐responsive supramolecular biomaterials: (32) disulfide group, (33) poly‐disulfide group, (34) succinimide–thioether group, (35) N‐mercapto‐based group (H_2_S donor), (36) S‐nitroso‐N‐acetylpenicillamine (NO donor). R1 and R2 = cytotoxic agent, other functional group, or macromolecule. (B) Schematic illustration of the response and immune mechanisms of sequence‐responsive multifunctional supramolecular nanomicelles. Adapted with permission [145]. Copyright 2023, WILEY‐VCH Verlag GmbH & Co. KGaA, Weinheim. (C) The structure of the GSH‐responsive supramolecular prodrug and its application to amplify photothermal immunotherapy of metastatic cancer. Adapted with permission [146]. Copyright 2024, Elsevier. (D) The preparation and anticancer mechanism of a NIR discrete metallacycle. Adapted with permission [147]. Copyright 2022, WILEY‐VCH Verlag GmbH & Co. KGaA, Weinheim. (E) A schematic illustration of a “dual lock‐and‐key” supramolecular photosensitizer. Adapted with permission [148]. Copyright 2020, Royal Society of Chemistry.

Redox reactions, which regulate the delicate balance between various oxidants and antioxidants, are among the most pivotal biochemical processes in living organisms. GSH, as a representative antioxidant, counteracts oxidative stress and protects lipids, proteins, and DNA. The significantly higher GSH concentrations in cancer cells provide a superior opportunity for constructing GSH‐responsive supramolecular biomaterials. In the GSH‐response process, the hydrogen from the thiol group of GSH is donated within the supramolecular biomaterial, enabling its reduction–oxidation conversion (GSH–glutathione disulfide) (Figure 6A). In the engineering of GSH‐sensitive supramolecular biomaterials, GSH‐cleavable functional groups such as disulfide bonds (─S─S─) [149], diselenide bonds (─Se─Se─), tetrasulfide bonds (─S─S─S─S─), 2‐mercaptopropionic acid ester moieties [150], imine bonds [151], and succinimide–thioether groups are typically incorporated as linkers or cross‐linking agents [152]. In particular, diselenide bonds are of lower bond energy than disulfide bonds and are responsive to both GSH and ROS [153]. This feature of diselenide bonds enables the design of more sensitive GSH‐responsive supramolecular systems; it also implies that their lower stability might lead to cargo leakage before they reach the tumor site. Hence, diselenide bonds are utilized primarily in ROS‐sensitive systems [154]. Moreover, succinimide–thioether moieties respond to GSH via Retro–Michael addition, thiol exchange reaction, and hydrolytic ring‐opening. As more complex reduction‐sensitive chemical structures, they have been little explored in GSH‐reactive supramolecular biomaterials, although there is limited literature on this subject [155].

In GSH‐sensitive supramolecular systems, therapeutic reagents such as drugs, genes, targeting groups, and imaging agents can either be chemically conjugated to host or guest molecules [156], the surfaces of (hybrid) nanoparticles [157] and the main or side chains of polymers [158]; or be physically encapsulated in the core, pore, or cavity of the cross‐linked micelle, nanogel, or liposome [159]. For instance, a multifunctional supramolecular nanomicelle PolyMN‐TO‐8 was self‐assembled from three components, comprising the host MTX‐MPEG2000, featuring enzyme‐sensitive carboxylate ester bonds; the prodrug NPX‐2S, incorporating a GSH‐responsive disulfide bridge; and the AIEgen TO‐8. This assembly was driven by host–guest and hydrophobic interactions. PolyMN‐TO‐8 could trigger pyroptosis‐assisted immunogenic cell death under laser irradiation while avoiding the risk of immune dysregulation. This strategy elicited robust antitumor immunity and tumor eradication via synergistic chemotherapy, PDT, and PTT (Figure 6B) [145]. In another example, an amphiphilic small molecule composed of a GSH‐sensitive linker, the hydrophobic immune checkpoint blocker NLG919, and the photothermal agent Cy7‐TCF was synthesized. It self‐assembled into a supramolecular prodrug via π–π stacking and hydrophobic interactions. In tumor cells, NLG919 was released upon GSH‐triggered disulfide bond cleavage to potentiate antitumor immunity. Subsequently, Cy7‐TCF reassembled into nanoparticles upon binding to endogenous albumin, which amplified its photothermal conversion and activated NIR fluorescence, thereby achieving synergistic photothermal immunotherapy against metastatic cancer (Figure 6C) [146].

Multivalent metal ions can be employed to prepare GSH‐responsive supramolecular biomaterials [160]. Reduced metal ions can further participate in Fenton/Fenton‐like reactions to advance the development of multi‐responsive and multimodal nanotherapeutic platforms. Common metal ions include Fe(III)/Fe(II), Cu(II)/Cu(I), Mn(IV)/Mn(II), and Pt(IV)/Pt(II) [161, 162]. Besides, several nitric oxide and hydrogen sulfide donors can potentially be utilized to develop GSH‐activated gas therapy [163]. Supramolecular biomaterials with 2‐azidomethyl benzoyl glycerol methacrylate (AZMB) are also sensitive to reducing environments and have been utilized in imaging‐guided radio‐chemotherapy for cancer treatment. As a representative example, a supramolecular coordination complex (MNP) was prepared by encapsulating a metallacycle within a GSH‐induced amphiphilic diblock copolymer (RGD‐POEGMA‐b‐PAZMB). The metallacycle was constructed from the perylene diimide (PPy) fluorophore and the tetraphenyldiplatinum (II) (TPE‐Pt) organometallic precursor. The reduction‐triggered cascade elimination of AZMB groups drove reversal of copolymer amphiphilicity, promoting release of the metallacycle. TPE‐Pt served as a dual‐modal agent for chemotherapy and radiosensitization, boosting the therapeutic efficacy, whereas PPy functioned as an NIR fluorescent probe, enabling real‐time treatment visualization (Figure 6D) [147]. Furthermore, a rare GSH‐sensitive group, a chlorine‐substituted BODIPY (BCl) fragment (Figure 6E), was introduced into a “dual lock‐and‐key” supramolecular photosensitizer for enhanced PDT, wherein the chlorine of the BCl moiety could be substituted with GSH to form a hydrophilic molecule [148].

### 3.4 ROS‐Responsive Supramolecular Biomaterials

**FIGURE 7:**
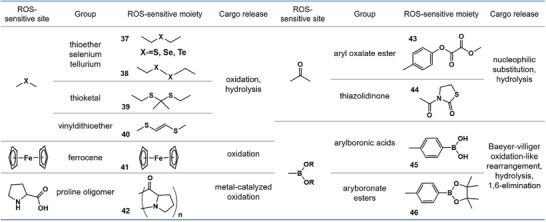
Representative functional groups and linkers employed for the design of ROS‐responsive supramolecular biomaterials.

Oxidation‐sensitive supramolecular biomaterials are designed to regulate redox homeostasis. On demand, they can generate ROS to potentiate oxidative stress for enhanced cancer therapy; conversely, they can be programmed to scavenge excessive ROS, alleviating oxidative damage and mitigating the inflammatory cascade. The mechanisms of ROS‐responsive supramolecular biomaterials can be attributed to ROS‐triggered structural cleavage and ROS‐triggered non‐cleavable hydrophobic–hydrophilic transition [164]. In oxidation‐sensitive biomaterials containing ROS‐sensitive chemical motifs such as thioketals, phenylboronic acids/esters, vinyl dithioethers, amino acrylate, oligoproline, and peroxalate ester, oxidative conditions can ultimately lead to structural disruption and scaffold collapse [165, 166]. In contrast, in oxidation‐sensitive biomaterials containing chalcogen elements such as sulfur [167], selenium [168], tellurium [169], or organometallic compounds such as ferrocene [170], oxidative conditions can promote the transformation of the supramolecular structures from hydrophobic to hydrophilic states. During this process, these elements can be readily oxidized from divalent to tetravalent or hexavalent states via the formation of covalent oxygen–sulfur bonds and non‐covalent hydrogen bonds between the polarized groups and surrounding water molecules [171]; the hydrophobic ferrocene can be oxidized into hydrophilic ferricinium with a charge transition from neutral to cationic (Figure 7) [172]. In summary, ROS‐responsive supramolecular biomaterials—which can deliver various therapeutic payloads, including chemotherapy drugs, immune‐related reagents, and imaging reagents—integrate ROS‐reactive chemical groups into their building blocks and undergo self‐assembly with the help of supramolecular interactions.

**FIGURE 8:**
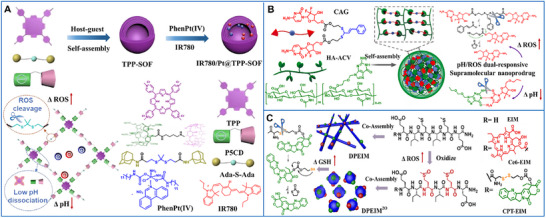
(A) The composition and response principle of pH/ROS dual stimuli‐responsive supramolecular organic frameworks for synergistic therapy. Adapted with permission [173]. Copyright 2023, Elsevier. (B) Schematic illustration of the preparation and antitumor mechanism of ROS‐activatable self‐amplifying supramolecular nanoprodrug for tumor‐specific therapy. Adapted with permission [174]. Copyright 2021, Elsevier. (C) Schematic illustration of ROS‐regulated self‐assembly of peptides into transformable scaffolds for cascade therapy. Adapted with permission [175]. Copyright 2021, Elsevier.

A dual‐responsive supramolecular organic framework (TPP‐SOF) was engineered through hierarchical orthogonal assembly of a porphyrin photosensitizer (TPP), a ROS‐responsive adamantane dimer (Ada‐S‐Ada), and a di‐macrocyclic host (P5CD). The framework was then co‐loaded with a chemotherapeutic prodrug (PhenPt(IV)) and a photosensitizer (IR780) via electrostatic and hydrophobic interactions, forming the nanoparticle IR780/Pt@TPP‐SOF. It disassembled through acidity‐mediated protonation of P5CD and ROS‐mediated breakage of the thioketal linker, exhibiting remarkable synergistic chemotherapy, PDT, and PTT effects under dual‐light irradiation (Figure 8A) [173].

ROS can also degrade thioacetal linkers—a novel class of ROS‐cleavable type—allowing scission and disassembly of supramolecular structures and producing ketone derivatives as byproducts. An ROS‐triggered supramolecular nanoprodrug was developed, comprising an ROS‐activated self‐immolative prodrug (CAG) and a valacyclovir‐modified hyaluronic acid conjugate (HA‐ACV) assembled via G≡C‐like hydrogen bonding. Within tumor cells, high ROS levels readily cleaved the thioacetal linker to release cinnamaldehyde and gemcitabine; the cinnamaldehyde then compensated for ROS consumption, establishing a self‐boosting “snowballing” effect that enhanced antitumor therapy (Figure 8B) [174].

Moreover, polypeptide chains containing proline, arginine, histidine, methionine, and lysine residues impart oxidation sensitivity and can serve as ROS‐responsive moieties. ROS‐responsive β‐sheet scaffolds (DPEIM) were prepared by co‐assembling two hexapeptide derivatives containing drug (CPT‐EIM) or photosensitizer (Ce6‐EIM). Laser irradiation‐generated ROS triggered a morphological transformation from nanofibers into nanoparticles. This rare ROS‐induced self‐assembly process was mediated by the hydrophobic‐to‐hydrophilic conversion of methionine residues within the polypeptides upon their oxidation from thioether to sulfoxide or sulfone forms (Figure 8C) [175].

The preparation of ROS‐responsive supramolecular systems involves not only endogenous ROS from NADPH enzyme or mitochondrial metabolism, but also exogenous ROS sources. For instance, in PDT, photosensitizers—representative exogenous‐ROS sources—excite oxygen to its singlet state under light irradiation. This requires careful alignment of the oxidation conditions of the chemical components with the ROS sources. Furthermore, ROS levels may also be up‐regulated via Fenton reactions [176], catalase‐mimic nanozymes [177], enzymatic reactions (e.g., those that utilize glucose oxidase and β‐lapachone) [178], and therapeutic reagents (e.g., succinate, α‐tocopheryl, and vitamin K3) [179], all of which can generate ROS.

### 3.5 ATP‐Responsive Supramolecular Biomaterials

**FIGURE 9:**
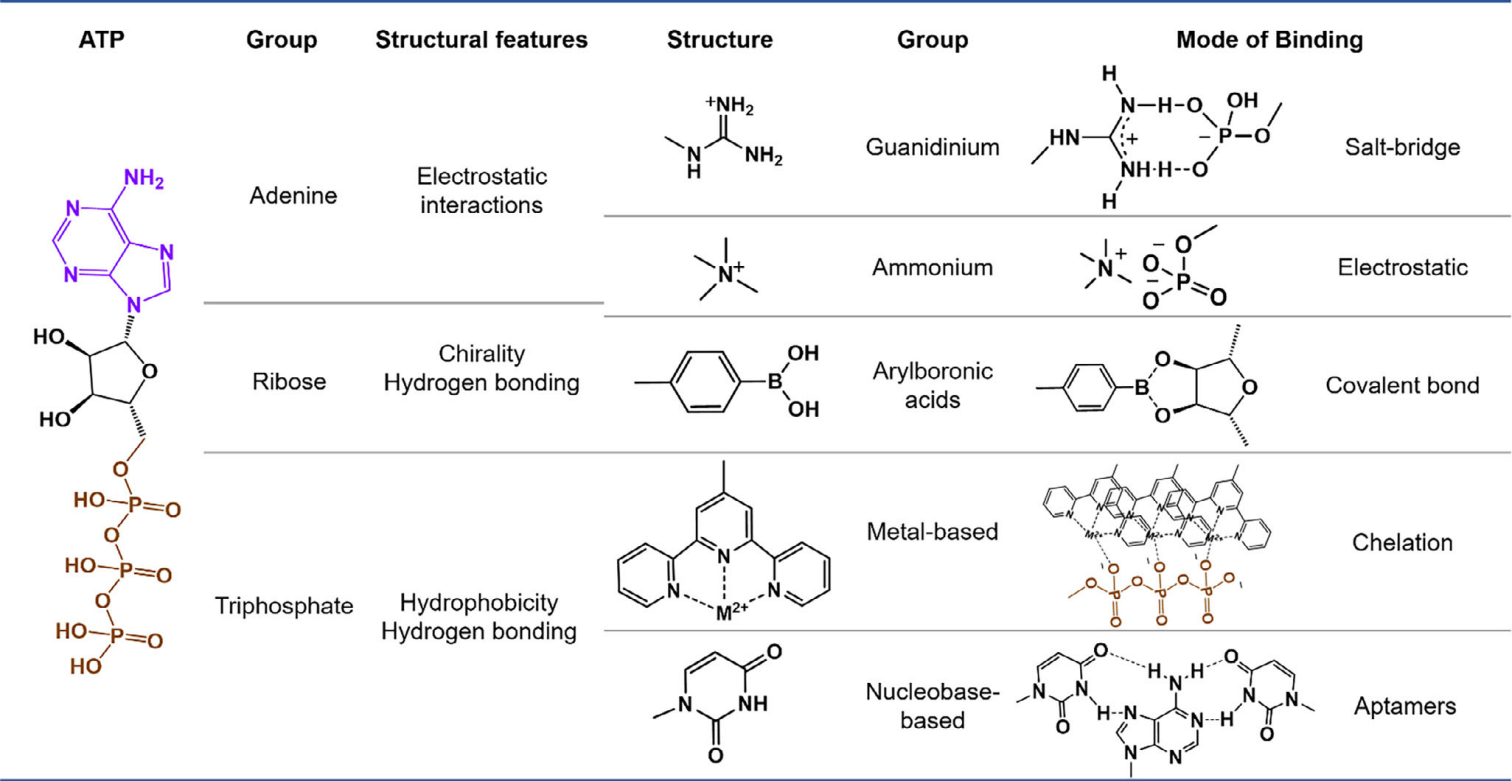
The composition and design principle of ATP‐responsive supramolecular biomaterials.

ATP acts as the molecular unit of currency in biological energy transfer, with its levels varying substantially between intracellular and extracellular spaces. This contrast has sparked interest in selecting ATP to build responsive supramolecular biomaterials for cancer treatment and theranostics. Specifically, ATP can be employed either as a co‐assembling building block or as a stimulus to elicit a response through the transfer part of its structure [180, 181]. In the former approach, the stimulus response is initiated by changes in molecule size, amount of charge, hydrophilic equilibrium of ATP, or the strength of the self‐assembly driving force (e.g., host–guest interaction, electrostatic interaction, π–π interaction, or hydrogen bonding) [182]. For instance, ATP could form dimers and even stacks through intermolecular association, as a result of loss of hydrophilicity upon further acidification; ATP could foster self‐aggregation through either specific bridging interactions or nonspecific charge screening, as a result of the presence and choice of additional salinity and counterions. In the latter approach, stimulus response is elicited by ATP binding to organic small‐molecule receptors or ATP‐specific aptamers integrated within the supramolecular biomaterial [183, 184]. For instance, phenylboronic acid binds specifically to the ribose moiety of ATP by formation of a covalent bond; biguanide binds specifically to the phosphate moiety of ATP by formation of a hydrogen bond [185]; xanthene binds specifically to the adenine moiety of ATP via π–π interactions; amino binds specifically to the phosphate moiety of ATP by formation of a salt‐bridge; and metal ion binds specifically to ATP by chelation (Figure 9) [186]. In short, ATP promotes either conformational change or structural disruption by competitively binding to the unloading site of the cargo. Given this, DNA/protein nanoassemblies, liposomes, hydrogels, microcapsules, supramolecular metal–organic frameworks, and supramolecular hybrid biomaterials thus act as cargo containers with an ATP lock [187]. In addition, ATP‐responsive supramolecular systems can also be engineered using enzymes that can hydrolyze ATP or react with ATP to form functional groups [188].

**FIGURE 10:**
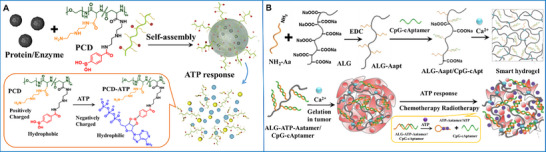
(A) The composition and response principle of ATP‐charged nanoclusters for cancer theranostics. Adapted with permission [189]. Copyright 2020, Elsevier. (B) Schematic illustration of the ATP‐induced preparation of smart hydrogel and the ATP‐triggered release of immune adjuvant for synchronization with repeated chemotherapy or radiotherapy to improve antitumor immunity. Adapted with permission [191]. Copyright 2021, WILEY‐VCH Verlag GmbH & Co. KGaA, Weinheim.

Significant progress has been achieved in the design of organic small‐molecule receptors capable of binding ATP for cancer therapy and diagnostics. As a representative example, ATP‐activated nanoclusters were prepared from a phenylboronic acid‐modified polycationic polymer (PCD) by encapsulating two enzymes through electrostatic and hydrophobic interactions. Intracellular ATP binds to the phenylboronic acid moiety of PCD via its pentose‐ring diols, rapidly reversing PCD charge and hydrophobicity and thereby disrupting the nanoclusters to release the enzymes. The ensuing enzymatic cascade induced starvation therapy and photoacoustic imaging (Figure 10A) [189]. Another example utilizes the ATP‐metal chelation mechanism for photo‐immunometabolic therapy. ATP‐depleted nanocomplexes (IR@ZIF‐RGD) were engineered via zinc(II)‐coordinated self‐assembly with imidazole‐2‐carboxaldehyde and an siRNA/polyethyleneimine complex, subsequently loaded with indocyanine green and coated with RGD‐modified polylactic acid‐hyperbranched polyglycerol. IR@ZIF‐RGD depleted ATP both by triggering framework degradation via strong zinc(II)‐ATP coordination and by restricting ATP synthesis via siRNA‐targeted antioxidant enzymes. IR@ZIF‐RGD also induced mitochondrial oxidative stress and dysfunction, thereby reprogramming tumor metabolism and reversing immunosuppression [190].

Aptamers, which are short sequences (∼ 30 bases) of single‐stranded DNA or RNA with high binding affinity and selectivity toward ATP [192], have been widely incorporated into ATP‐responsive nanoformulations for the delivery of diverse cargoes ranging from metal ions and small molecules to proteins [193]. For instance, an ATP‐responsive hydrogel was developed to elicit controlled immune responses for enhanced immunotherapy. This system was fabricated by Ca^2+^‐crosslinking an alginate‐conjugated ATP‐specific aptamer (ALG‐Aapt) with its complementary strand hybridized with the immune adjuvant CpG oligonucleotide (CpG‐cApt). A surge in ATP, stemming from dying tumor cells undergoing immunogenic cell death induced by low‐dose oxaliplatin or X‐rays, competitively bound to the aptamer. This binding displaced the CpG‐cApt, thereby disintegrating the hydrogel and promoting antitumor immunity (Figure 10B) [191].

### 3.6 Enzyme‐Responsive Supramolecular Biomaterials

All biological and metabolic processes are related to enzymes. Exploiting enzymes as triggers offers unique superiorities, including substrate specificity, exceptional selectivity, and effectiveness under mild conditions (neutral pH, low temperature, and buffered aqueous media), where many conventional chemical reactions fail. Many nanoscale materials, such as polymer‐based biomaterials (e.g., micelles, vesicles, nanoparticles, and hydrogels) [194], liposomes, peptide‐based materials [195, 196], and hybrid materials, have been utilized in the design of enzyme‐responsive supramolecular biomaterials [197, 198]. They adopt two key strategies. The first involves enzyme‐responsive destruction or modulation of supramolecular structural integrity, which mainly proceeds through processes such as substrate hydrolysis [199, 200], charge conversion, structural changes (e.g., from sphere to rod) [201], amphiphilic transformation [202], or sol–gel transition (e.g., from liquid‐to‐solid phase transition to liquid–liquid phase separation) [202, 203]. The second involves enzyme‐triggered self‐assembly, which relies on either the breaking of chemical bonds upon enzymatic hydrolysis or the formation of (non)covalent bonds upon enzymatic catalysis [62, 204, 205]. In view of this, enzyme‐responsive supramolecular biomaterials demonstrate enormous potential in cancer diagnosis, imaging, and treatment.

**FIGURE 11:**
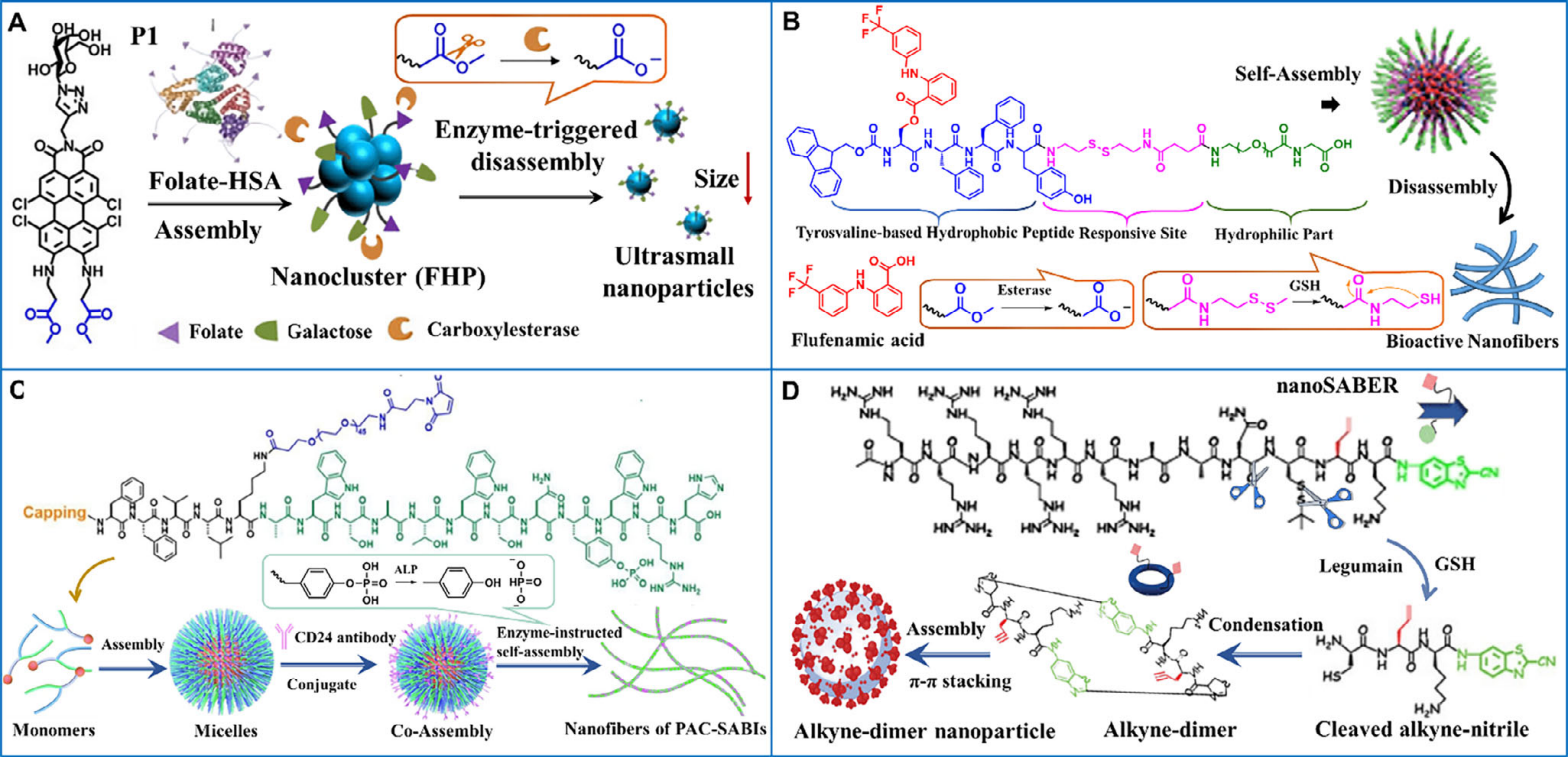
(A) Schematic illustration of the enzyme‐triggered disassembly of photosensitizer‐based nanoclusters for activatable and deep PDT. Adapted with permission [207]. Copyright 2020, WILEY‐VCH Verlag GmbH & Co. KGaA, Weinheim. (B) Schematic illustration of the preparation and response principle of supramolecular nanomedicine for triple‐negative breast cancer treatment. Adapted with permission [208]. Copyright 2022, American Chemical Society. (C) Schematic illustration of the preparation of ALP‐mediated self‐assembly for enhanced anticancer immunotherapy. Adapted with permission [209]. Copyright 2024, Springer Nature. (D) Schematic diagram of a self‐assembled bioorthogonal enzyme recognition (nanoSABER) probe with Raman activable ability. Adapted with permission [210]. Copyright 2023, WILEY‐VCH Verlag GmbH & Co. KGaA, Weinheim.

The most common stimuli‐responsive method of cargo release from different supramolecular biomaterials relies on site‐specific enzymatic cleavage [200, 206]. Carboxylesterase‐responsive nanoclusters (FHP) with deep‐tumor penetration were assembled from a tailored near‐infrared precursor (P1) and folate‐decorated albumin. The P1 contained a skeleton of tetrachloroperylene monimide functionalized with β‐alanine methyl ester and galactose, in which β‐alanine methyl ester served as both the enzyme‐cleavable motif and electron donor, and galactose served as the tumor‐targeting ligand and hydrophilic group. With the overexpression of esterase in the TME, the FHP shrank from 100 to 10 nm, accelerating deeper penetration, enhancing NIR fluorescence intensity and singlet oxygen generation, thereby enabling in situ imaging and promoting deep PDT performance (Figure 11A) [207].

An enzyme‐responsive peptide was engineered as a structural transition system for cancer therapy. The peptide, incorporating flufenamic acid and tyrosylvaline as hydrophobic moieties, polyethylene glycol as the hydrophilic moiety, and ester and disulfide bonds as cleavable linkers, was self‐assembled into “core–shell” shaped nanospheres. In response to the overexpression of esterase and GSH, these nanospheres transformed into fibrous structures and released encapsulated drugs. These drugs upregulated upstream Hippo pathway signals to stimulate protein phosphorylation and cytoplasmic degradation, while synergizing with downstream Hippo pathway signals to block protein–transcription factor interactions, thereby inhibiting the growth and metastasis of triple‐negative breast cancer (Figure 11B) [208].

Enzyme‐triggered self‐assembly of supramolecular biomaterials is an emerging field in anticancer therapy [211]. Among various self‐assembly driving forces, the dephosphorylation of phosphorylated precursors by alkaline phosphatases overexpressed in cancer cells has been the focus of extensive research [212, 213]. Similarly, matrix metalloproteinase‐responsive precursors self‐assemble upon interaction with matrix metalloproteinase [57, 214]. An alkaline phosphatase‐mediated in situ self‐assembly system was developed using a tailored peptide monomer (Pep‐PEG) to reactivate macrophages for enhanced immunotherapy. The Pep‐PEG was composed of a β‐amyloid‐derived peptide sequence functionalized with fluorophores, a hydrophilic PEG linker, and a CD47/SIRPα‐blocking peptide. Through sequential antibody modification and enzyme‐guided rearrangement, the Pep‐PEG was hierarchically assembled into nanofibers. These nanofibers effectively disrupted macrophage phagocytosis checkpoints, thereby potentiating antitumor immune responses (Figure 11C) [209]. The bioorthogonal probe was developed for multi‐scale tumor imaging by integrating Raman spectroscopy with enzyme‐induced intracellular self‐assembly. Its design incorporated a polyarginine cell‐penetrating sequence, a legumain recognition sequence, and a nitrile group from 2‐cyanobenzothiazole as the capping group. In response to legumain and GSH, the probe was self‐assembled into alkyne‐dimer nanoparticles. This transformation reduced the Raman peak intensity of the nitrile group, as it participated in the formation of a new thiazole ring during a click condensation reaction. This study established a direct causal relationship between enzyme activity and Raman signals (Figure 11D) [210].

### 3.7 Light‐Responsive Supramolecular Biomaterials

Light serves as a potent medium for the transfer of energy and information, indicating that light‐responsive supramolecular biomaterials play pivotal roles in diagnostic imaging, biomarker sensing, therapeutic delivery, PDT, and PTT [215, 216]. The most frequently utilized light sources include ultraviolet light (UV; λ, 100–315 nm), visible light (Vis; λ, 400–700 nm), and near‐infrared light (NIR; λ, 700–1750 nm). Chemiluminescence, electrochemical luminescence, and bioluminescence, has attracted widespread attention because of its superior penetration depth (∼ 13 cm for NIR vs. <2 mm for visible light), which greatly influences the theranostics efficacy for deep tissues and organs [217].

The various light sources possess exceptional features that are well‐suited for their respective applications [218]. Photoactivation can be achieved in three ways: (i) photochemical reactions (i.e., photo‐cleavage or photo‐isomerization) [219, 220], (ii) photosensitization [221, 222, 223], (iii) photothermal activation [70, 224]. For these purposes, photo‐responsive components must be (non‐)covalently incorporated into supramolecular biomaterials. Three classes of light‐responsive motifs dominate current photobiomaterials. Photolabile cleavable groups—typified by nitrobenzyl and coumarin derivatives—undergo irreversible bond scission upon irradiation [225]; photo‐isomerizable switches—azobenzene, diarylethene, spiropyran, and stilbene—reversibly interconvert between stereoisomers without releasing by‐products [226]; photoactive agents—fluorophores‐based materials, polymers, transition‐metal complexes, and inorganic nanomaterials—simultaneously enable imaging‐guided drug delivery and generate ROS or heat for PDT and PTT [227]. For example, fluorophores include porphyrins, methylene blue, indocyanine green, benzo[*c*]thiophene, boron dipyrromethene, cyanine, squaraine, and perylene bisimide [228]; polymers include polydopamine [39]; transition metal complexes include cyclometalated Ir(III) complexes and Ru(II) polypyridyl complexes [229, 230]; and inorganic nanomaterials include gold nanoparticles and upconverting nanoparticles [231].

**FIGURE 12:**
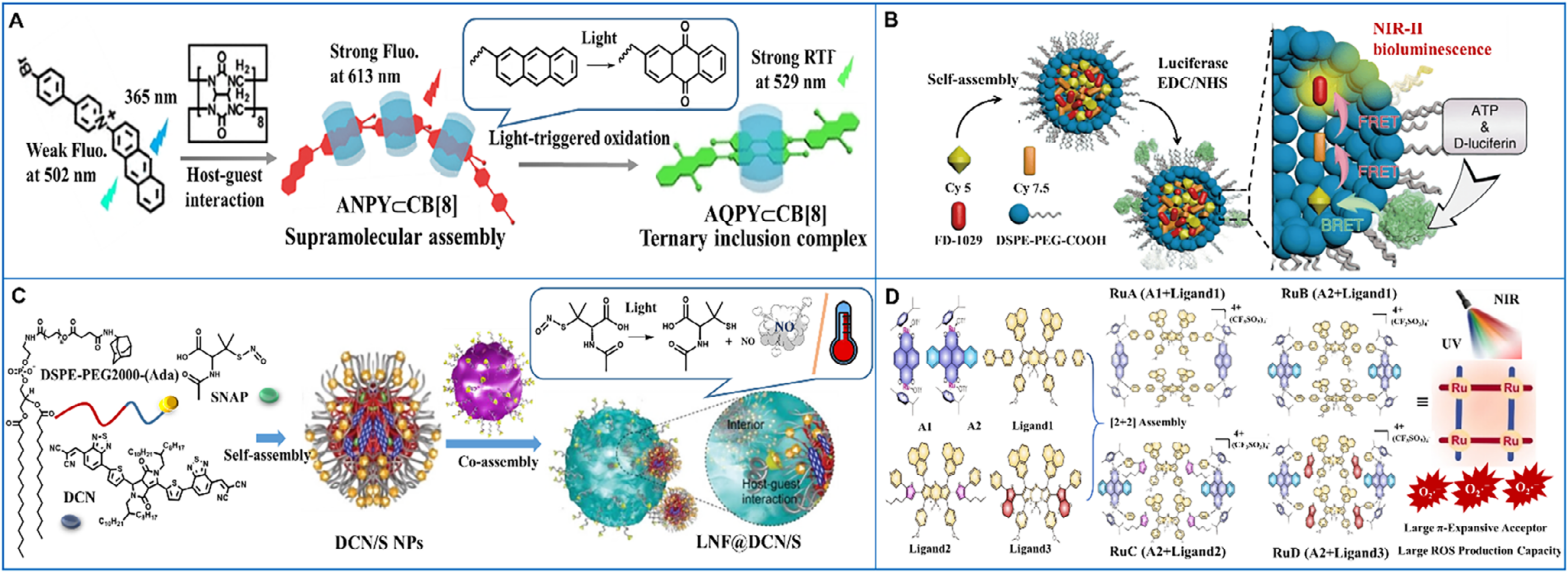
(A) Schematic illustration of a photooxidation‐driven luminescent conversion from supramolecular assembly for lysosome‐targeted imaging. Adapted with permission [234]. Copyright 2021, American Chemical Society. (B) The composition and working mechanism of NIR‐II triggered bioluminescence probes (BPs). Adapted with permission [235]. Copyright 2020, Springer Nature. (C) Schematic illustration of the triple combination of PTT, gas therapy, and immunotherapy via supramolecular cell‐conjugation drug delivery platform to trigger the robust antitumor immune response. Adapted with permission [236]. Copyright 2023, WILEY‐VCH Verlag GmbH & Co. KGaA, Weinheim. (D) Schematic illustration of the preparation and the antitumor mechanisms of the engineered metallacycle‐based supramolecular photosensitizer for effective PDT. Adapted with permission [237]. Copyright 2023, WILEY‐VCH Verlag GmbH & Co. KGaA, Weinheim.

Light can be harnessed in large‐scale photochemical reactions that transform light energy into chemical bonding energy or induce conformational variations [232, 233]. As a representative example, an anthracene‐based photo‐oxidation reaction was employed as the building block to construct a supramolecular assembled entity with rationally tunable photoluminescence for dual‐organelle imaging of lysosomes and nuclei in living cells. Upon interaction with the cucurbit[8]uril (CB[8]), the anthracene‐conjugated bromophenylpyridinium guest (ANPY) formed a “head‐to‐tail” supramolecular polymer (ANPY⊂CB[8]) that emitted red fluorescence due to the host‐stabilized intermolecular charge‐transfer interactions. Subsequently, UV irradiation induced a structural transformation to a “head‐to‐head” homoternary inclusion (AQPY⊂CB[8]) that emitted green phosphorescence as a result of extensive halogen‐bonding interactions (Figure 12A) [234].

Endogenous light sources, which are promising luminescence sources, have also been employed in the construction of supramolecular biomaterials. An NIR‐II bioluminescence probe was designed to achieve 1029 nm emission through a relay of bioluminescence resonance energy transfer and two‐step fluorescence resonance energy transfer. The probe was constructed from luciferase‐conjugated micelles encapsulating a blend of specialized and commercial dyes. This design enabled high‐contrast bioimaging of vasculature and lymphatics, achieving a 5‐fold higher signal‐to‐noise ratio and a 1.5‐fold greater spatial resolution than conventional methods (Figure 12B) [235].

Light‐responsive supramolecular biomaterials for PDT have garnered significant attention over the past few decades, as they can be converted from a steady‐state to an excited state under light irradiation to achieve selective cytotoxicity. Thus far, considerable efforts have been devoted to developing photosensitive materials to overcome the drawbacks of insufficient penetration depth, poor spatial selectivity, and oxygen‐dependence [238, 239, 240]. Transition‐metal complexes can facilitate intersystem crossing from singlet to triplet states through heavy‐atom effects. For instance, a series of Ru(II) metallacycle‐based supramolecular photosensitizers (RuA–RuD) was prepared for effective PDT. These structures self‐assembled from π‐expansive Ru‐based acceptors and specially designed aza‐boron‐dipyrromethene ligands bearing strong donor groups (julolidinyl and anisole groups) and ruthenium coordination sites (phenylpyridine, 3‐butylthiophene, or 3,4‐ethylenedioxythiophene). Among them, metallacycle RuD exhibited high NIR phototoxicity, strong absorption and emission, and exceptional hypoxic‐tolerance, owing to its minimal singlet‐triplet energy gap and maximal steric hindrance (Figure 12D) [237].

The photothermal effect is often associated with the photo‐triggered process of supramolecular biomaterials. Ideally, a photothermal biomaterial should have high photothermal conversion efficiency while minimizing thermal damage to the surrounding healthy tissue [241]. One way to improve PTT efficacy is to promote the distribution of photothermal agents in the internal tumor tissue, where the photosensitizer exerts the largest agglomerative thermal effect. As an illustration, a novel diketopyrrolopyrrole‐based photothermal reagent (DCN) was reported in a supramolecular cell‐conjugation platform (LNF@DCN/S) for synergistic therapy. The DCN and a nitric‐oxide donor were co‐encapsulated in an adamantane‐modified polymer to obtain nanoparticles (DCN/S), which were rapidly anchored onto β‐cyclodextrin‐decorated liquid‐nitrogen‐frozen cancer cells (LNF) via host–guest interactions. The resulting LNF@DCN/S improved tumor penetration through activation of matrix metalloproteinases —a process induced by peroxynitrite, which was derived from heat‐induced nitric oxide release; LNF@DCN/S boosted tumor immunotherapy via heat‐triggered immunogenic cell death, thereby achieving PTT‐gas therapy‐immunotherapy for tumor eradication (Figure 12C) [236].

### 3.8 Temperature‐Responsive Supramolecular Biomaterials

Even small temperature fluctuations can profoundly affect the activity of biological systems, and well‐controlled temperature increases are beneficial in cancer treatment. The application of supramolecular biomaterials in PTT, ultrasound‐mediated thermal therapy, and magnetic hyperthermia has been comprehensively reviewed elsewhere and hence is not incorporated in this discussion.

**FIGURE 13:**
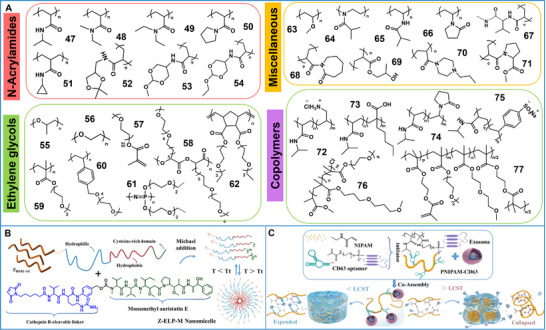
(A) Chemical structures of common temperature‐sensitive components (homo‐ and co‐polymers). N‐substituted acrylamide based polymers: (47) PNIPAM, (48) PEMAM, (49) PDEAM, (50) PAPy, (51) PNcPAM, (52) PDMDOMA, (53) PNMM, (54) PNEM; ethylene glycolbased polymers: (55) PPO, (56) PEO(PEG), (57) OEGMA_1100_, (58) OEOPL, (59) PMEO_2_MA, (60) P(TrEGSt), (61) PBEEP, (62) POEGNB; miscellaneous polymers:(63) PMVE, (64) PiPOx, (65) PNVIBA, (66) PVPy, (67) PAVMA, (68) PNVCL, (69) PHPA, (70) PNANPP, (71) P(A‐Pro‐OMe); copolymers: (72) PNIPAM‐co‐PAH, (73) PNIPAM‐co‐PBMA, (74) PNIPAM‐co‐PNVP, (75) PNIPAM‐co‐PSS, (76) P[MEO_2_MA_90_‐co‐(OEGMA_500_)_10_], (77) P[(DEGMA‐ME)‐co‐OEGMA‐ME_475_‐co‐EGDMA]. (B) Formation of the thermal‐responsive nanomicelle for actively targeted treatment of ovarian cancer. Adapted with permission [253]. Copyright 2024, American Chemical Society. (C) Schematic illustration of aptamer‐modified temperature‐responsive polymer for early detection of bladder cancer through efficient urine exosome enrichment. Adapted with permission [254]. Copyright 2024, Elsevier.

Thermal‐responsive supramolecular biomaterials currently used for cancer theranostics are typically (hybrid) polymer nanoparticles, micelles, and hydrogels that have an upper or lower critical solution temperature (UCST or LCST, respectively) (Figure 13A) [242, 243, 244]. Their conformation and properties are susceptible to ambient temperature. When the temperature rises above the UCST, UCST‐type biomaterials undergo a hydrophobic‐to‐hydrophilic transition, dissociating or reassembling into smaller aggregates and simultaneously release their loaded cargo. UCST temperature‐sensitive components include poly(N‐acryloylglycinamide) (PNAGA) and poly(acrylamide‐co‐acrylonitrile) (P(AAm‐co‐AN)) [245]. In contrast, when the temperature rises above the LCST, LCST‐type biomaterials adjust the conformation from hydrophilic to hydrophobic without disassembly, which drives the cargo to be expelled outward. LCST temperature‐sensitive elements include poly[2‐(2‐methoxyethoxy)ethyl methacrylate] (PMEO_2_MA) [246], poly(amidoamine) (PAMAM) [247], poly(N‐vinylcaprolactam) (PNVCL) [248], poly(2‐oxazoline)s (POxs) [249], poly(N‐isopropylacrylamide) (PNIPAM) [250], poly(oligo(ethylene glycol)methacrylate) (POEGMA) [251], and polypeptide‐based polymers [252].

An LCST‐type nanomicellar platform was developed for hyperthermia‐activated ovarian cancer therapy. The platform employed a peptide‐drug conjugate (Z‐ELP‐M) based on thermoresponsive elastin‐like polypeptides, which were functionalized with targeting affibodies via protein fusion and conjugated to a cytotoxic drug via Michael addition. When the temperature exceeded the conjugate's phase‐transition point, Z‐ELP‐M immediately self‐assembled into micelles. This thermal response promoted selective drug accumulation and effective tumor targeting at physiological temperatures for superior antitumor efficacy (Figure 13B) [253]. Similarly, another temperature‐responsive system was designed for noninvasive early diagnosis of bladder cancer by capturing and separating urinary exosomes. The system consisted of poly(N‐isopropylacrylamide) (PNIPAM) decorated with exosome‐specific aptamers (PNIPAM‐CD63). Below the LCST, the dispersed network specifically trapped exosomes via aptamer–protein interactions; above the LCST, it collapsed into dense spherical structures and subsequently isolated exosomes from the solution owing to hydrogen bond rupture. This approach showed rapid isolation efficiency (within 5 min) and significantly improved sensitivity, especially for early‐stage and low‐grade bladder cancers (Figure 13C) [254].

Liposomes are perhaps the most promising thermal‐responsive supramolecular biomaterial, as they are safe and prone to respond to tiny temperature fluctuations. Liposome‐based drugs have already been administered in several clinical trials for cancer therapy [255]. The thermo‐sensitive components in liposomes can contain 1,2‐distearoyl‐sn‐glycero‐3‐phosphocholine (DSPC), 1,2‐dipalmitoyl‐sn‐glycero‐3‐phosphocholine (DPPC), 1,2‐dipalmitoyl‐sn‐glycero‐3‐phosphoglyceroglycerol (DPPGOG), and other materials [256]. A thermal‐responsive liposomal nanoagent (PCT) was developed by co‐encapsulating two therapeutic agents and a semiconducting polymer within a DSPE‐PEG and DPPC lipid bilayer. At the phase‐transition temperature of 41 °C, the thermo‐responsive DPPC membrane was destroyed, allowing the activated release of drugs. Such superposition therapy effectively ablated primary tumors and suppressed metastasis [257].

### 3.9 Ultrasound‐Responsive Supramolecular Biomaterials

Ultrasound is known for its non‐invasiveness, cost‐effectiveness, and safety [258], enabling it to function as the “initiator” and “remote control” of intelligent supramolecular systems [259]. It exhibits excellent performance in tumor diagnostic and therapeutic purposes based on its thermal, mechanical, and chemical effects. In some cases, these mechanisms occur simultaneously and must be studied as a whole rather than in isolation. These biological effects can be elicited by modulating ultrasound intensity and frequency. Specifically, an increment in intensity can drive a controlled amount of energy to the target site, and an elevation in frequency can enhance the depth of penetration and likelihood of cavitation [260].

**FIGURE 14:**
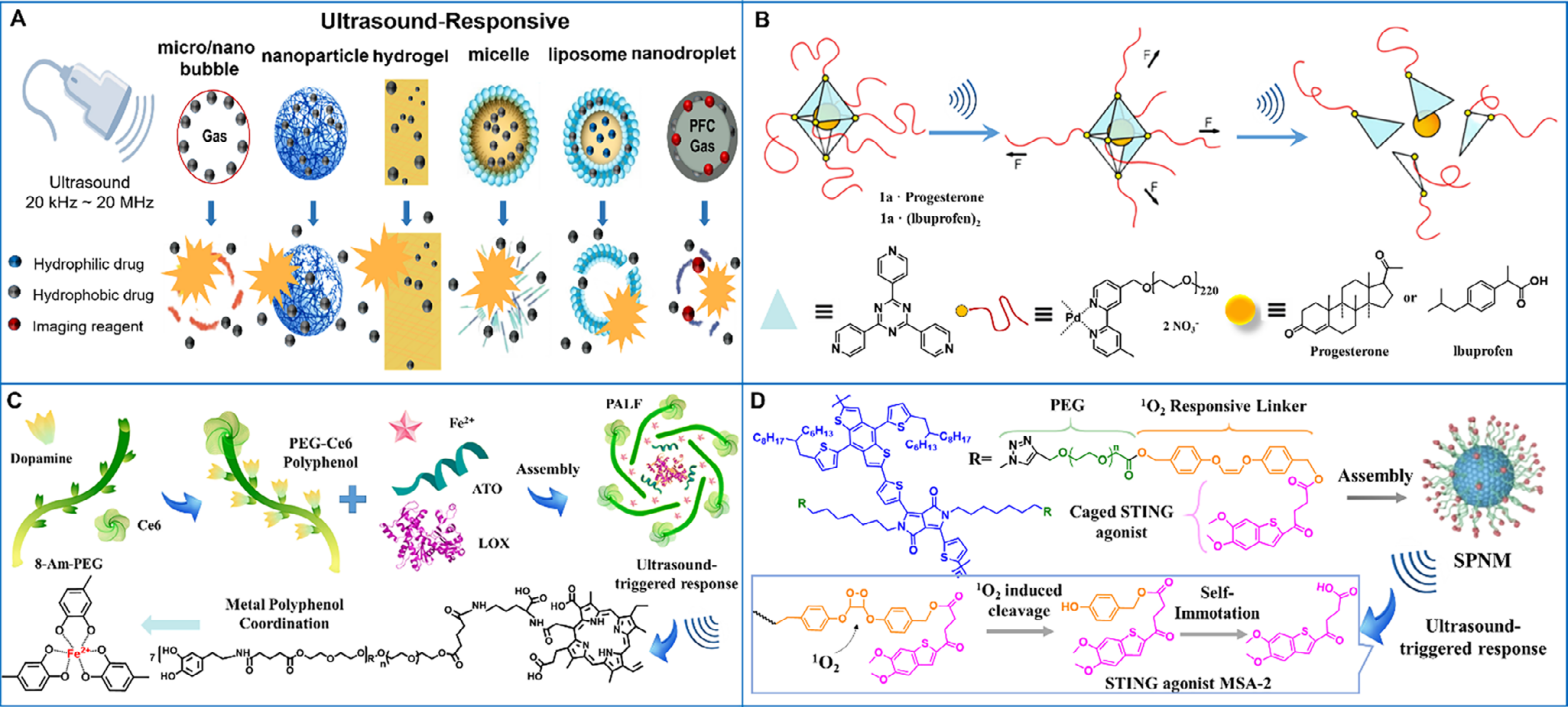
(A) A schematic illustration of the structure of ultrasound‐responsive material for cancer theranostic procedures. (B) Schematic illustration of the shear force produced by ultrasonication for drug release. Adapted with permission [272]. Copyright 2021, WILEY‐VCH Verlag GmbH & Co. KGaA, Weinheim. (C) Schematic illustration of the functional chemical structures and fabrication process of the metal‐phenolic network‐based nanocomplex for SDT. Adapted with permission [273]. Copyright 2021, American Chemical Society. (D) Schematic illustration of sono‐driven STING activation using semiconducting polymeric sonosensitizers for cancer immunotherapy. Adapted with permission [274]. Copyright 2023, WILEY‐VCH Verlag GmbH & Co. KGaA, Weinheim.

Converting acoustic energy into other forms is heat. The absorption of high‐intensity focused ultrasound by a specific focal target causes local hyperthermia that either destroys the targeted cancer cells or triggers the release of the payload from the thermo‐sensitive structure [261]. Converting acoustic energy into other forms is mechanical energy. The pressure changes caused by propagating ultrasonic waves generate mechanical effects, such as shear stress, that can either activate drugs by scissoring covalent and perturbing non‐covalent intermolecular bonds [262, 263] or enhance endocytosis by shearing or stretching the cell membrane to form transient holes [264]. Converting acoustic energy into other forms is chemical energy. The energy from the combination of ultrasound and catalytic biomaterials (sonosensitizers) initiates or accelerates various redox catalytic reactions that produce abundant reactive radicals (·O_2_^−^, ·OH, ^1^O_2_) under complex living conditions. This process is described as “sonocatalytic therapy” [265]. While this review focuses on supramolecular systems, for completeness, inorganic systems are also summarized in this section. Ultrasound‐responsive supramolecular (hybrid) materials include polymer‐based systems (e.g., microbubbles, dendrimers, hydrogels, micelles) [266], biomimetic‐based systems (e.g., liposomes) [267], perfluorocarbon‐based phase‐change nanodroplets [268], ROS‐producing systems [269], and non‐polymer‐based systems (e.g., gold nanoparticles, superparamagnetic iron oxide nanoparticles, and mesoporous silica nanoparticles) [270, 271]. They can act through at least one of the three response mechanisms (Figure 14A).

Conversion of ultrasound to heat energy is primarily applied to tumor thermal ablation therapy, which is one of the FDA‐approved cancer treatments. It has also been validated that ultrasound‐mediated heating can elicit immune reactions by generating immunogenic debris [275]. Considering the unexpected damage to the surrounding normal cells from long‐term hyperthermia, ultrasound‐mediated thermotherapy has been investigated as a delivery system for precise drug activation [276]. Meanwhile, ultrasound‐induced mechanical effects are also employed to ensure spatiotemporally precise release of cargo by scissoring the covalent bondsand disrupting non‐covalent interactions within macromolecular frameworks, including covalent bonds such as those in tension rings, isomerization bonds, and bonds with low dissociation energy (e.g., peroxide (O–O), disulfide (S–S), and ester bonds), as well as non‐covalent interactions such as coordination reactions [277, 278]. For instance, in an ultrasound‐controlled delivery platform based on the self‐assembled supramolecular cage Pd^II^_6_(TPT)_4_, therapeutic agents such as progesterone and ibuprofen were loaded into its hydrophobic nanocavity and released upon the collapse of its star‐shaped structure mediated by ultrasonic shear force. This ultrasound‐activated, non‐covalent strategy represented a promising approach for hypotoxic cancer therapy (Figure 14B) [272]. Aside from drug activation, there are also many studies on ultrasonically triggered natural supramolecular systems loaded with proteins or nucleic acids, providing a blueprint for the field of sonogenetics through mechanically mediated remote control [279, 280].

Sonodynamic therapy, the most typical candidate of sonochemical effects, primarily focuses on ROS generation from the interaction between sonosensitizers and low‐intensity focused ultrasound. The sonosensitizers are excited by ultrasound or heat to generate electron–hole (e^−^/h^+^) pairs, which escape from the material surface and catalyze the substrate to promote ROS generation in an aqueous environment [281]. These ROS can induce cell apoptosis, necrosis, or pyroptosis, and can activate prodrugs through a cascade of chemical and biological events. For instance, a nanocomplex based on metal–phenolic networks was developed for enhanced sonodynamic therapy. It was constructed through coordination and hydrophobic interactions among a chlorin e6‐conjugated polyphenol sonosensitizer, ferrous ions, lactate oxidase, and the mitochondrial inhibitor atovaquone. It functioned by synergistically generating toxic ROS, depleting intracellular lactate, and reducing oxygen consumption, thereby effectively inhibiting tumor proliferation and metastasis (Figure 14C) [273].

The sonodynamic approach has been extended to enable precision immunotherapy of head‐and‐neck squamous cell carcinoma. A nanoagonist (SPNM) was self‐assembled using a sonodynamic semiconducting polymer backbone conjugated to the non‐nucleotide stimulator of interferon genes (STING) agonist (MSA‐2) via a ROS‐cleavable linker. Upon ultrasound irradiation, SPNM generated substantial ROS, which not only initiated immunogenic cell death but also sheared the linker to release MSA‐2 for in situ STING activation. This sono‐driven STING activation promoted dendritic cell maturation and cytotoxic T lymphocyte infiltration, inhibiting tumor growth and establishing long‐term immunological memory (Figure 14D) [274].

### 3.10 Magnetic‐Responsive Supramolecular Biomaterials

Magnetic therapy, which utilizes artificially generated static or alternating magnetic fields, is a clinically recognized method for diagnosing, preventing, and treating cancers. Candidates for magnetic‐responsive supramolecular biomaterials rely on magnetically active (bio)materials, including iron oxide nanoparticles (Fe_3_O_4_, Fe_2_O_3_), iron oxide‐based hybrid nanoparticles (e.g., with transition metals (Co, Ni, Ag, Pt, Au) or rare earth metals (La)), magnetic metal‐doped materials (Cu, Mn, Gd) [282], magnetic proteins [283], and bacterial magnetic particles [284], which possess common magnetic properties such as ferromagnetism, ferrimagnetism, antiferromagnetism, and (super)paramagnetism. These magnetic cores are decorated with prefabricated polymer chains on their exterior through (non)covalent bonding; these magnetic cores are appended on or encapsulated in pre‐engineered cell membranes or well‐constructed layered structures via supramolecular interactions (e.g., hydrophobic and host–guest interactions) [285]; these magnetic elements self‐assemble with organic ligands to form supramolecular structures via coordination interactions [286]. The exterior surface of the magnetic cores is modified to enhance their biocompatibility, shield them from the surrounding environment, extend the circulation time in the body, and enrich the anchoring sites for the attachment of functional modules.

Various magnetically guided supramolecular biomaterials, including vesicles [287], liposomes [288], polymeric micelles [289], polymeric nanoparticles [290], hydrogels [291], supramolecular self‐assemblies [292], metal–organic frameworks [293], have been architected. Prominent applications of magnetic‐responsive supramolecular biomaterials in cancer therapy include magnetically controlled delivery, magnetic‐mediated hyperthermia therapy, magnetic‐assisted dynamic therapy, and magnetic resonance imaging [294, 295, 296]. In addition, such materials hold promising potential in cancer immunotherapy, as they destroy tumor cells via magnetically induced mechanical vibrations and regulate the immunological TME by triggering bursts of toxic ROS, thereby reprogramming tumor‐associated macrophage polarization and T cell infiltration into the tumor [297].

The effective loading, targeted transport, and controlled release of therapeutic cargo are pivotal goals in cancer therapy. Magnetically driven delivery is emerging as a potential therapeutic delivery mechanism. Various agents, including chemotherapeutic drugs, immune adjuvants, antibodies, inhibitors, nucleic acids, and antioxidant enzymes, have been integrated into magnetic reservoirs by conjugation, surface coating, or encapsulation. When a static or alternating magnetic field is applied externally, it first assists the targeting of the drug carrier and then opens the “gate” or “channel” of the drug carrier by inducing structural alterations via magnetic forces or magneto–thermal effects. These alterations include rearrangement of crosslink networks, rupture of self‐assembled scaffolds, compression of micelles, disintegration of magnetic cores, and hydrogel swelling.

**FIGURE 15:**
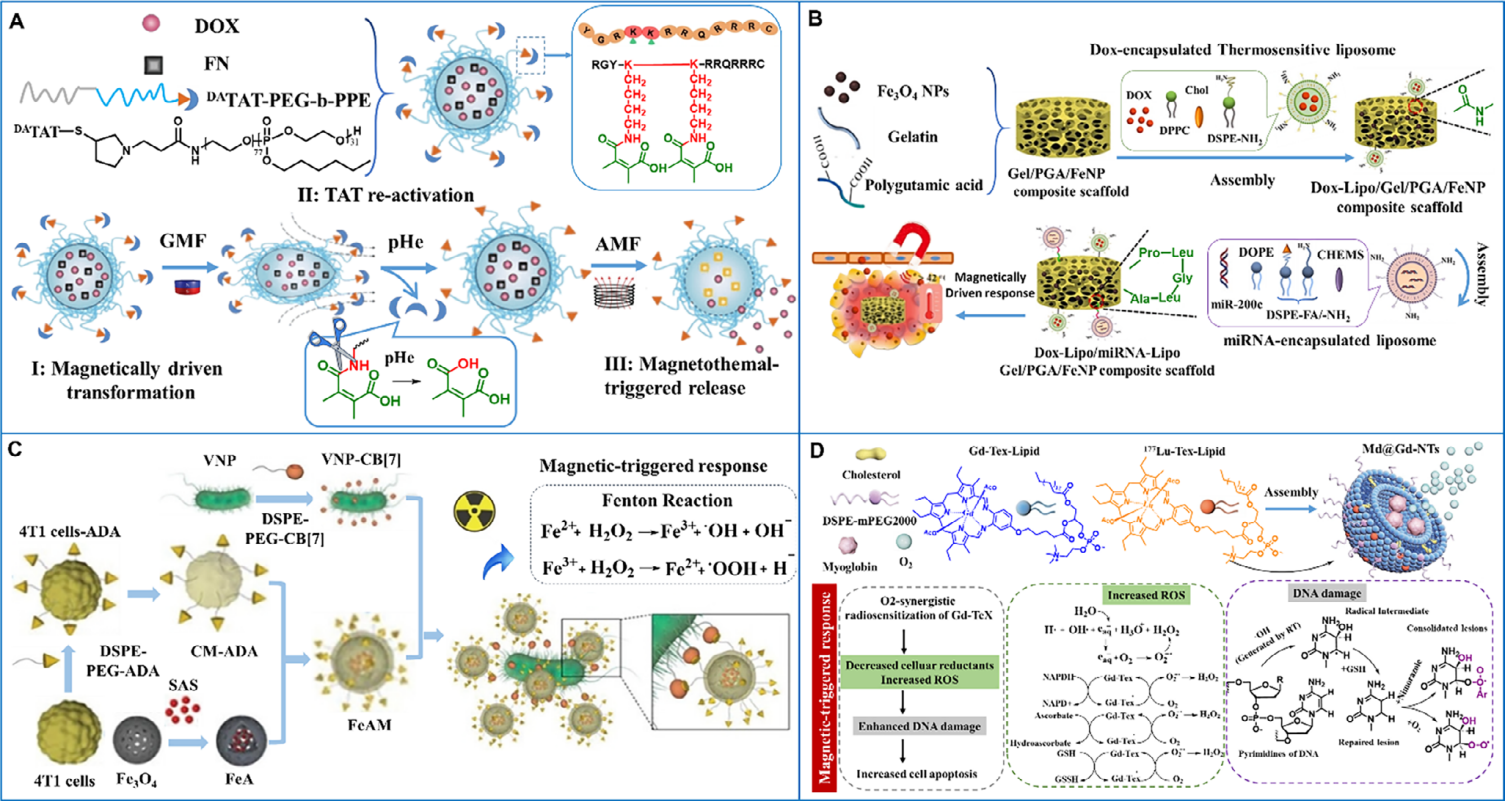
(A) Schematic illustration of a magnetically driven nanorobot (amNR) for whole‐process active drug delivery. Adapted with permission [298]. Copyright 2023, WILEY‐VCH Verlag GmbH & Co. KGaA, Weinheim. (B) The composition of magnetic nanoparticle‐based composite scaffold loaded with Dox‐liposomes and miRNA‐liposomes, and their working mechanism of synergistic magnetic thermal therapy and chemotherapy. Adapted with permission [300]. Copyright 2025, Elsevier. (C) Schematic illustration of supramolecular ferroptosis inducers via triple integration of living bacteria, cell membranes, and magnetic nanoparticles to enhance antitumor immunotherapy. Adapted with permission [301]. Copyright 2023, WILEY‐VCH Verlag GmbH & Co. KGaA, Weinheim. (D) Schematic illustration of the fabrication and the antitumor mechanism of Gd^3+^‐coordinated texaphyrin liposome‐like nanovesicles (Mb@^177^Lu/Gd‐NTs) from supramolecular assembly for MRI‐guided radiosensitization therapy. Adapted with permission [302]. Copyright 2023, Springer Nature.

A representative platform for magnetic‐driven drug delivery was the amoeba‐like nanorobot (amNR), which was engineered by functionalizing a copolymer with an acid‐sensitive targeting peptide and loading it with ferrimagnetic nanocubes and doxorubicin. The amNR operated through a multistep mechanism to achieve deep tumor penetration via magnetic field‐driven deformation, activate cellular uptake by acidic TME‐induced ligand exposure, and trigger magneto‐thermal drug release under an alternating magnetic field, thereby inhibiting tumor growth (Figure 15A) [298]. Although magneto–thermal‐triggered release is a valuable approach, it is also feasible to use magnetic forces to control drug release [299].

Magnetic‐responsive supramolecular biomaterials also unveil a landscape of opportunity for application in hyperthermia therapy. They can function as transducers under pulsed or alternating high‐frequency magnetic fields to convert hysteresis or relaxation loss (Néel or Brownian) into heat, thereby exposing tumor cells to elevated temperatures and leading to cellular thermally induced apoptosis and necrosis [303]. Unlike traditional clinical thermal modalities, magnetic‐mediated hyperthermia is applied directly at the tumor tissue level. Specifically, the nanoheaters are anchored within tumor cells to facilitate tumor‐killing effects in a highly selective, precise, and low‐toxicity manner. For instance, a porous scaffold was fabricated from magnetic nanoparticles (Fe_3_O_4_ NPs), gelatin, and polyglutamic acid using an ice microparticle‐mediated hybridization method. To this scaffold, doxorubicin‐loaded thermosensitive liposomes and microRNA‐loaded enzyme‐cleavable liposomes were anchored via coupling and click reactions. The resulting composite specifically released microRNA for sensitization chemotherapy and generated potent magnetic hyperthermia upon irradiation, whereas doxorubicin release was synchronized with the hyperthermia effect, enabling a synergistic therapeutic approach against drug‐tolerant breast cancer (Figure 15B) [300].

ROS‐based dynamic therapy is a cancer therapeutic modality to achieve endogenous‐ or exogenous‐triggered ROS production in the tumor region. Magnetic‐mediated dynamic therapy, one form of dynamic therapy, can effectively promote tumor regression by increasing intracellular ROS levels [304, 305]. Magnetically assisted ferroptosis and tumor immunotherapy are exemplary illustrations. A magnetite‐mediated supramolecular conjugate (FeAMV) was prepared through host–guest self‐assembly between cucurbit[7]uril conjugated with living bacteria and an adamantane‐embedded cell membrane wrapping sulfasalazine‐loaded Fe_3_O_4_ nanoparticles. The resulting FeAMV exhibited excellent tumor‐specific accumulation due to bacterial colonization, cell membrane homing effects, and magnetic targeting. Furthermore, the Fe_3_O_4_ nanoparticles and sulfasalazine synergistically initiated ferroptosis through the Fenton reaction, effectively inhibiting tumor growth and potentiating immunotherapy (Figure 15C) [301].

In addition, magnetic‐responsive supramolecular biomaterials can be employed as contrast agents in magnetic resonance imaging to facilitate early diagnosis and real‐time monitoring of treatment effects [306]. For instance, lipid bilayer nanovesicles (Gd‐NTs) were self‐assembled from “texaphyrin‐lipids” building blocks chelated with Gd^3+^ and ^177^Lu^3+^ ions. After encapsulating the oxygen‐carrying protein myoglobin, the resulting platform (Mb@Gd‐NT) served as an imaging‐guided radiosensitization reagent. It provided single‐photon emission computed tomography/magnetic resonance imaging dual‐modality imaging through the coordinated metal ions and achieved radiosensitization through spatiotemporally precise oxygen delivery, thereby demonstrating clinical potential for imaging‐guided radiotherapy (Figure 15D) [302]. Mn^2+^‐chelate complexes have been approved as magnetic resonance imaging contrast agents in preclinical research. The coordination‐driven self‐assembled Mn(II)‐risedronate‐manganese nanobelts (RMn‐NBs) were investigated for magnetic resonance imaging, in which the Mn^2+^ ions also acted as adjuvants to activate the cGAS‐STING signaling pathway, thereby boosting cancer immunotherapy [307].

## 4 Significant Challenges in the Clinical Application

The diversity of supramolecular interactions has significantly facilitated the design of biomaterials with sophisticated architectures. The dynamic and reversible nature of supramolecular interactions has endowed outstanding stimuli‐responsive properties, showing immense potential for cancer diagnosis and therapy. In particular, multifunctional materials enable intelligent targeting, controlled cargo delivery, and specific activation of antitumor pathways in a stimuli‐responsive manner via physiological (pH, O_2_, GSH, ROS, ATP, and enzyme) or external stimuli (light, temperature, ultrasound, and magnetic fields). Together, these strategies can contribute to the development of multimodal synergistic cancer therapies and the advancement of existing treatments, ultimately improving patient outcomes while minimizing treatment‐related adverse effects.

Although remarkable progress in supramolecular biomaterial‐based therapeutics has been made in the laboratory, their clinical translation continues to be hindered by design‐related, technological, or biological constraints. There are two key design challenges. (a) Greater insight is required into the self‐assembly mechanisms of supramolecular biomaterials in water. Solute–solvent interactions substantially affect the properties of supramolecular biomaterials. In aqueous solutions, (co)self‐assembly is typically driven by hydrophobic collapse rather than directional interactions [308], which contributes to the complexity of the assembly mechanism. (b) Supramolecular interactions may reduce the stability of nanoplatforms to some extent. In particular, biomaterials injected intravenously into the bloodstream should remain stable in complex biological systems. Balancing weak and strong interactions remains a crucial hurdle.

The technological development of supramolecular biomaterials is challenging on various fronts. (a) Despite the precise control over small‐scale production of biomaterials in the laboratory, their large‐scale synthesis under the guidelines of good manufacturing practice remains limited. (b) Several parameters of biomaterials, such as storage shelf‐life and quality assurance, are also important factors that affect therapeutic efficiency. (c) Animal models commonly employed in preclinical research, mainly mouse models, might not accurately predict clinical responses and often encounter challenges in replicating in vivo results observed in human trials. These factors are essential to ensure the clinical success of supramolecular biomaterials.

Finally, three critical biological challenges exist in developing these biomaterials. (a) Some natural products and biopolymer derivatives are promising building blocks for supramolecular biomaterials. However, the design strategies often impair their innate biocompatibility and biodegradability. (b) The localization and penetration of supramolecular biomaterials at tumor sites remain a tough task. Supramolecular biomaterials should pass serially through physical and physiological barriers, which are complex systems including several layers (e.g., intravascular barriers, endothelial barriers, extracellular barriers, and cellular barriers) and multiple components (e.g., intratumoral pressure, dense extracellular matrix, enzymatic barriers, endosomal compartments, and mononuclear phagocytic system) [309]. (c) The biosafety and degradability of supramolecular biomaterials are prerequisites for their biomedical application and clinical transformation. Therefore, it is imperative to conduct in‐depth investigations of their complex toxicity profiles and metabolic effects to guarantee their safety and sustainability in human trials.

In addition to the shared clinical translation challenges of supramolecular biomaterials—design‐related, technological, and biological barriers—stimuli‐responsive biomaterials face unique translational hurdles. Typical self‐assembled supramolecular biomaterials, such as protein‐drug co‐assemblies (Abraxane, albumin‐bound paclitaxel nanoparticle), liposomes (Doxil, liposomal‐polyethylene glycol loaded with doxorubicin; Onivyde, liposome loaded with irinotecan), polymer microspheres (Eligard, PLGA polymer loaded with leuprolide acetate), and micelles (mPEG‐PLA loaded with paclitaxel) [310, 311], have achieved tremendous clinical success. In contrast, the clinical translation of stimuli‐responsive supramolecular biomaterials remains unsatisfactory, as evidenced by the failure of ThermoDox (thermosensitive DOX‐loaded liposomes, Phase III clinical trial, 2021), LE‐SN38 (enzyme‐responsive camptothecin prodrug‐loaded liposomes, Phase II clinical trial, 2021), and ProLindac (pH‐sensitive HPMA‐platinum polymer micelles, Phase II clinical trial, 2011). Visudyne, a liposomal formulation of verteporfin for photodynamic therapy, has emerged as a successful clinical treatment option in ophthalmology.

Indeed, exploiting supramolecular self‐assembly principles to develop stimuli‐responsive anticancer biomaterials remains a novel field, and many challenges remain in bringing them from the bench to the bedside. Different types of stimuli‐responsive supramolecular biomaterials are confronted with characteristic translational hurdles that must be addressed individually. For pH‐responsive biomaterials, the urgent demand is that they should bypass the recognition of opsonins (proteins involved in macrophage recognition and immune response activation) in the plasma; otherwise, they would be taken up and cleared by the reticuloendothelial system before achieving therapeutic efficacy. For redox‐ and enzyme‐responsive biomaterials, the major concern is their insufficient selectivity for efficient treatment, owing to the overlapping spectra of redox conditions and the actions of enzymes within both healthy and tumor tissues. For ATP‐responsive biomaterials, the important consideration is to avoid interference from guanosine triphosphate or even other nucleoside (tri)phosphates. For light‐responsive biomaterials, the critical drawback is their limited signal penetration and light‐focusing capability within deep tissue regions. For temperature‐responsive biomaterials, the significant challenge is the out‐of‐sync action time due to temperature gradients at the tumor site. Finally, for ultrasound‐ and magnetic‐responsive biomaterials, the crucial bottleneck is the need for specialized equipment to provide ultrasound waves and external magnetic fields.

While not all studies in this domain require immediate translatability, the deliberate integration of utility‐oriented design principles remains critical. (1) Multi‐scale cross‐attention methodology: this approach quantifies how supramolecular properties arise from cross‐scale interactions (molecular to macroscopic), simultaneously disentangling dominant intra‐scale interactions and their inter‐scale coupling dynamics mechanism. (2) Logic‐gate design: logic operations are essential for building orthogonal controllable systems that can be activated by specific molecular inputs via bio/chemical reactions, bio‐catalysis, bio‐affinity, or multilevel supramolecular interactions. (3) “Self‐immolative” prodrug monomers design: the drug itself integrates into the supramolecular motif, so disassembly spontaneously releases the active pharmaceutical ingredient without residual carrier toxicity. (4) Beyond “EPR effect‐only” strategies: the synergistic combination of active targeting moieties (including peptide ligands, aptamers, or antibodies) and stimuli‐responsive release systems achieves superior therapeutic precision. (5) Imaging‐drug co‐assemblies design: this strategy permits real‐time biodistribution monitoring and adaptive dosing control.

## 5 Conclusion and Future Perspective

Supramolecular interactions, more akin to a versatile toolkit of reversible, dynamic, and directional capabilities, bring a lot of convenience and flexibility to obtain attractive biomaterials with ordered architectures, tailored morphologies, and multiple loaded cargoes. Furthermore, the introduction of physiological or extracorporeal stimuli enables these sophisticated supramolecular biomaterials to offer spatiotemporal control for applications ranging from bioimaging to cancer theranostics. This review summarizes the latest process of cancer theranostics based on diverse supramolecular biomaterials: (1) supramolecular polymer‐based materials, (2) biomimetic‐based materials, (3) supramolecular coordination complexes, (4) supramolecular hybrid materials, and (5) supramolecular nanovalves. These biomaterials act differently toward indispensable stimuli: acidic environment, hypoxia, disrupted redox equilibrium, elevated ATP levels, and enzyme overexpression in the TME, as well as light, temperature, ultrasound, and magnetic fields. These stimuli can induce variations in the size, charge amount, hydrophobic–hydrophilic equilibrium, and mechanical strength of supramolecular biomaterials through physical or chemical changes, or a combination thereof. The response mechanisms include (i) the introduction of sensitive groups that can undergo protonation, deprotonation, cleavage of chemical linkages, formation of (non)covalent bonds, valence changes of metal ions, and solubility changes without bond cleavage, mainly in response to physiological stimuli; (ii) the integration of chemical building blocks that respond to extracorporeal stimuli. While responsive‐stimuli supramolecular materials have been extensively investigated in the laboratory, their clinical applications still face several challenges.

To advance the new generation of cancer theranostics with clinical application potential, several key areas merit exploration. It is necessary to consider carefully supramolecular biomaterials that respond specifically to tumor‐associated subcellular compartments [312], such as the lysosomes or mitochondria, thereby optimizing therapeutic outcomes while minimizing off‐target effects. Incorporating cutting‐edge applications of artificial intelligence, such as machine learning and deep learning, computer‐aided design of intelligent stimuli‐responsive supramolecular biomaterials would further enhance the efficacy and safety of their clinical translation [313]. Exploiting patient‐compatible stimuli‐responsive supramolecular biomaterials for personalized treatment will drive unprecedented clinical progress in cancer theranostics.

## Conflicts of Interest

The authors declare no competing financial interests.
